# Modeling Contextual Modulation of Memory Associations in the Hippocampus

**DOI:** 10.3389/fnhum.2018.00442

**Published:** 2018-11-09

**Authors:** Praveen K. Pilly, Michael D. Howard, Rajan Bhattacharyya

**Affiliations:** Center for Human-Machine Collaboration, Information and Systems Sciences Laboratory, HRL Laboratories Malibu, CA, United States

**Keywords:** hippocampus, contextual coding, pattern completion, dentate gyrus, memory encoding, memory retrieval, memory interference, catastrophic forgetting

## Abstract

We present a computational model of how memories can be contextually acquired and recalled in the hippocampus. Our adaptive contextual memory model comprises the lateral entorhinal cortex (LEC), the dentate gyrus (DG) and areas CA3 and CA1 in the hippocampus, and assumes external inputs about context that originate in the prefrontal cortex (PFC). Specifically, we propose that there is a top-down bias on the excitability of cells in the DG of the hippocampus that recruits a sub-population of cells to differentiate contexts, independent of experienced stimuli, expanding the “pattern separation” role typically attributed to the DG. It has been demonstrated in rats that if PFC is inactivated, both acquisition and recall of memory associations are impaired. However, PFC inactivation during acquisition of one set of memory associations surprisingly leads to subsequent facilitation of the acquisition of a conflicting set of memory associations in the same context under normal PFC operation. We provide here the first computational and algorithmic account of how the absence or presence of the top-down contextual biases on the excitability of DG cells during different learning phases of these experiments explains these data. Our model simulates PFC inactivation as the loss of inhibitory control on DG, which leads to full or partial activation of DG cells related to conflicting memory associations previously acquired in different contexts. This causes context-inappropriate memory traces to become active in the CA3 recurrent network and thereby the output CA1 area within the hippocampus. We show that these incongruous memory patterns proactively interfere with and slow the acquisition of new memory associations. Further, we demonstrate that pattern completion within CA3 in response to a partial cue for the recall of previously acquired memories is also impaired by PFC inactivation for the same reason. Pre-training the model with interfering memories in contexts different from those used in the experiments, simulating a lifetime of experiences, was crucial to reproduce the rat behavioral data. Finally, we made several testable predictions based on the model that suggest future experiments to deepen our understanding of brain-wide memory processes.

## 1. Introduction

A defining characteristic of our daily lives is our ability to recall memories of experiences that occurred in arbitrary places, even from the distant past. So, a fundamental question is what enables the brain to represent these seemingly countless memories? It has been known for a long time that the hippocampus is critical for the formation of long-term declarative memories. Its main function is to rapidly bind together multi-modal cortical signals representing a current event into a memory engram (Poo et al., 2016), such that a partial cue presented later can trigger the reactivation of the corresponding engram and thereby the episodic memory recall (Marr, 1971; Squire and Alvarez, 1995). Distributed regions beyond the medial temporal lobe, such as the prefrontal cortex (PFC), are also known to be involved in memory processing, but their roles are computationally less clear. In this regard, the main goal of this article is to advance a computational account of how external contextual signals can modulate various aspects of associative memory encoding and recall in the entorhinal-hippocampal system. In support of our computational model, we present simulation results that replicate experimental data from rats related to regulating interference in the encoding and recall of context-based associative memories. That data showed that memory associations can be not only impaired but also facilitated by the inactivation of PFC under various conditions (Navawongse and Eichenbaum, 2013; Peters et al., 2013). In particular, if PFC is inactivated, both acquisition and recall of memory associations are impaired. However, PFC inactivation during acquisition of one set of memory associations surprisingly leads to subsequent facilitation of the acquisition of a conflicting set of memory associations in the same context under normal PFC operation. Furthermore, the typical advantage for learning a conflicting set of memory associations in a different context is lost under PFC inactivation.

Context is a high-level functional concept, more than simply a particular grouping of spatial locations. A set of items that need to be memorized together can signify a context, and so can a common set of task rules governing behavior in a given situation (Preston and Eichenbaum, 2013). In the Navawongse and Eichenbaum (2013) and Peters et al. (2013) experiments, contextual cues are supplied by placing the rats in rooms with different wallpapers or ambient odors that serve to functionally distinguish the varied memory-based responses. When a familiar context is recognized, PFC is thought to bias the retrieval of context-appropriate memories in the hippocampus to guide current behavior (Preston and Eichenbaum, 2013). PFC is generally agreed to be the brain region responsible for top-down executive control, able to filter out arbitrary task-irrelevant neural representations in distributed cortical areas with its widespread connectivity (Miller and Cohen, 2001). Several studies support the involvement of PFC in memory processing, with coordinated interactions between PFC and hippocampus (Siapas and Wilson, 1998; Jones and Wilson, 2005; Peyrache et al., 2009; Kim et al., 2011; Brincat and Miller, 2015).

It is well known that different subsets of hippocampal neurons are assigned to encode memories experienced in different contexts (Kubie and Muller, 1991; Leutgeb et al., 2004, 2005). Specifically, DG neurons encoding various memories in one context are not only sparse and distributed but also statistically independent from those encoding memories in a different context (Markus et al., 1995; Doboli et al., 2000; Chawla et al., 2005; Liu et al., 2012; Ramirez et al., 2013), and involve a local inhibitory circuit for context-based retrieval (Raza et al., 2017). Our model makes a direct link between these two sets of data, positing coordinated interactions between PFC and DG for contextualizing memories. In particular, we demonstrate that the experimental data from the PFC inactivation experiments can be simulated by adding an external biasing signal over DG granule cells to exert inhibitory control of their excitability, whereby an independent subset of cells are recruited for the acquisition and recall of memory associations experienced in a given context. Based on the Navawongse and Eichenbaum (2013) and Peters et al. (2013) experiments, we propose that this external biasing signal is present only when the PFC is online. We believe our computational work adds to the emerging understanding of brain-wide memory processes at the neural, circuit, and network levels.

## 2. Model

**Figure 1:**
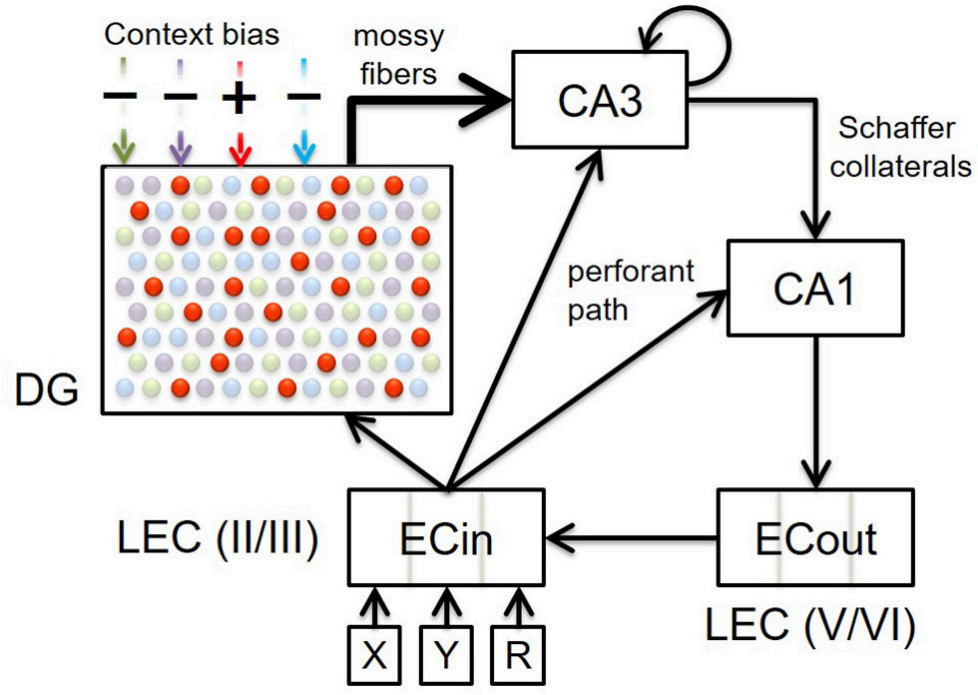
Block diagram illustrating the connectivity of our model. Top-down context bias modulates memory encoding and retrieval in the entorhinal-hippocampal system by dynamically facilitating only one subset of DG granule cells for a particular context (represented by red dots in this particular illustration). Here four contextual ensembles of DG cells are depicted, but note that there would be innumerable such ensembles within a real DG that are each recruited for a specific context. The superficial (II/III) and deep (V/VI) layers in only the lateral entorhinal cortex (LEC) are included, because the constituents of episodic memories relevant to the PFC inactivation experiments that our model simulates (Navawongse and Eichenbaum, 2013; Peters et al., 2013) are non-spatial odor cues. The connectivity within the entorhinal-hippocampal system follows well-known anatomical details: perforant path projections from superficial layers of LEC to DG, CA3, and CA1; powerful mossy fiber projections from DG to CA3; recurrent collaterals within CA3; Schaffer collaterals from CA3 to CA1; back-projections from CA1 to deep layers of LEC; and intracortical feedforward projections within LEC from deep to superficial layers. High-level cortical signals conveying requisite codes for the two odor cues in each discrimination problem arrive at LEC as the input. Memory retrieval of the odor associated with reward, for a given pair of cues, is assessed by comparing the activity in the deep layers of LEC at the end of the second half of the *minus* phase to the target pattern.

Our memory model (see Figure 1) provides a computational account of how episodic memory associations can be encoded and retrieved in a context-sensitive manner. It employs rate-coded point neurons in multiple layers and subfields within the entorhinal-hippocampal system; namely, superficial (ECin) and deep (ECout) layers of lateral entorhinal cortex (LEC), dentate gyrus (DG), and areas CA3 and CA1 within the hippocampus. CA3 is modeled as a dense recurrent neural network where active cells comprising the current episodic experience learn to rapidly “auto-associate” (i.e., learn projections to themselves), which allows for pattern completion of the corresponding “engram” pattern when only a subset of them are activated subsequently. The lateral entorhinal cortical signals traverse the hippocampal circuitry from LEC to CA1 along two streams; namely, the direct (monosynaptic) pathway: ECin → CA1, and the indirect (trisynaptic) pathway: ECin → (DG and CA3), DG → CA3, CA3 → CA1 (Amaral and Witter, 1995). Our model builds on a previous hippocampal model proposed by Ketz et al. (2013) in the *emergent* neural network simulator (O'Reilly and Munakata, 2000; Aisa et al., 2008). The Ketz model differentially modulates these hippocampal pathways in different phases of the hippocampal theta rhythm as suggested by some prior computational models (Hasselmo et al., 2002; Kunec et al., 2005); see Douchamps et al. (2013) for pertinent data.

We have made several changes to the Ketz et al. (2013) model. Our key innovation is to instantiate different subsets of DG cells in different situational contexts. This is critical as it relates to our main hypothesis that the contextual recruitment of DG cells is governed by a top-down inhibitory control mechanism. We also made a number of technical changes to conform to known anatomy and physiology. First, synaptic plasticity in a majority of the connectivity (perforant path: ECin → {DG and CA3}; mossy fibers: DG → CA3; Schaffer collaterals: CA3 → CA1) is fully Hebbian (i.e., *k*_*hebb*_ = 1.0 in Equation 6), and not a combination of Hebbian and error-driven learning that is heavily biased toward the latter, as in Ketz et al. (2013). Second, there are no EC-like “slots” (or groupings of cells) in CA1 coding individual input streams, as CA1 has conjunctive/episodic cells like CA3 (Leutgeb et al., 2004) that can develop and sustain even without CA3 inputs (Brun et al., 2002) in response to divergent perforant path projections from superficial layers of entorhinal cortex (Naber et al., 2001). Lastly, we removed ECout → CA1 backprojections, as there is little supporting evidence. Details of the model are described below in the Model Equations section.

## 3. Experimental details

We now briefly describe how a contextual memory association is formed and recalled in our model. Suppose a rat enters context *A* for the first time and encounters two dishes *X*_1_ and *Y*_1_ with distinct odors. The experimenter has placed a reward only in one dish, say *X*_1_, and the rat is allowed to dig for the reward only from one dish in each trial. If by chance the rat chooses the baited dish and thereby obtains the reward, then an episodic memory is formed for context *A* that is composed of the association between the two odor cues of *X*_1_ and *Y*_1_ and the presence of reward in *X*_1_ (i.e., {*X*_1_, *Y*_1_} → *X*_1_). As the memory of the rewarded event gets strengthened from several trials in context *A*, the rat gradually makes fewer errors in making the memory-guided choice whenever it encounters odor cues *X*_1_ and *Y*_1_ in context *A*. The rat can learn several other discrimination memories within the same context; e.g., {*X*_2_, *Y*_2_} → *X*_2_, {*X*_3_, *Y*_3_} → *X*_3_, and so on. In Experiment 1A of Peters et al. (2013) the rats were presented with a sequence of eight discrimination problems in random order on each day until they reached a criterion level of performance in terms of the number of correct choices across the eight pairs. The rats can also learn and remember a new set of odor discrimination problems in another context *B*. The context *B*-specific memories can exhibit different levels of overlap in the components of episodes from context *A*. In the experiment of Navawongse and Eichenbaum (2013), the rats were presented with the same pair of odor cues in context *B* (e.g., {*X*_1_, *Y*_1_}) but the baited dish was the opposite of that in context *A*; i.e., {*X*_1_, *Y*_1_} → *Y*_1_. And in Experiments 2 and 3 of Peters et al. (2013), the rats were required to learn a new set of odor discrimination problems either within the same context or a different context. Here the memories overlapped only in one component. In particular, one odor in each pair was retained and the reward prediction of this odor was reversed compared to the first set. For example, for the episode {*X*_1_, *Y*_1_} → *X*_1_ , if *X*_1_ were retained then the new rewarded event would be {*X*_1_, *Z*_1_} → *Z*_1_; whereas if *Y*_1_ were retained then the new rewarded event would be {*Z*_1_, *Y*_1_} → *Y*_1_.

**Figure 2:**
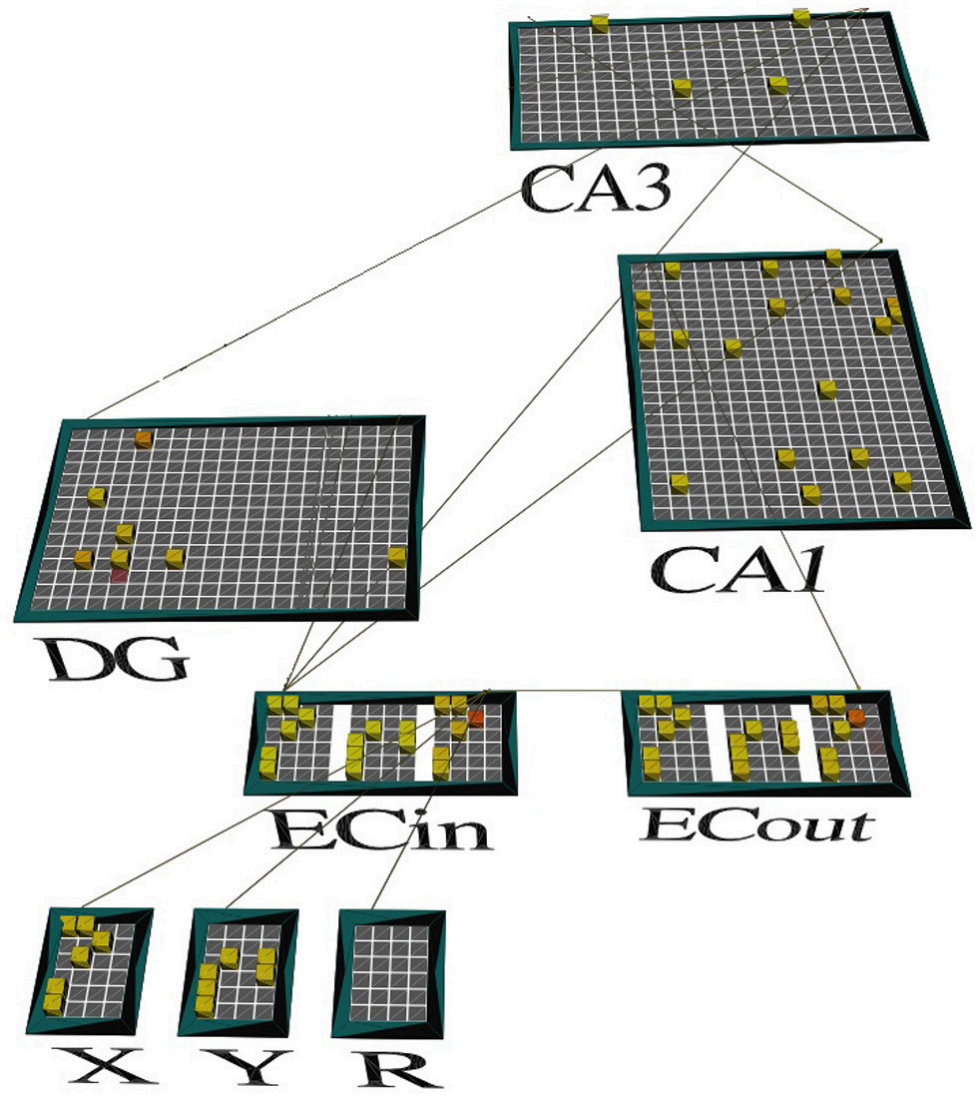
A screenshot of our model's activity. The network is shown in the *emergent* neural network simulator (Aisa et al., 2008) when exposed to context *A* at the end of the second half of the *minus* phase. Only the context *A*-relevant subset of DG cells (represented by the green dots in Figure 1) is facilitated to potentially become active (shown), with cell excitability drastically reduced for the other pools related to the other three (3) contexts (not shown). The odor stimuli that define the discrimination problem are represented by high-level neural codes in the segmented cortical pools named *X* and *Y*, upstream of the superficial layers of LEC (ECin). The cortical pool named *R* identifies which of the two odors is associated with reward in this problem. The memory system must complete the missing pattern for *R* in the corresponding pool (or slot) within the deep layers of LEC (ECout) based on intrahippocampal interactions, including those in CA3 recurrent network. The pattern recalled for *R* is the same as the one for *X*, which means that *X* is the one that led to the reward earlier.

The three components of each odor discrimination memory (e.g., {*X*_1_, *Y*_1_} → *X*_1_) are represented by sparse distributed codes in distinct cortical populations upstream of the entorhinal-hippocampal system; see groups of neurons labeled *X*, *Y*, and *R* in Figure 2. In our simulations, each unique odor is assigned a randomly constructed binary bit pattern on a 6x4 grid with exactly six cells turned on (maximal activation of 1). Our model incorporates four independent contextual subsets of cells in DG (namely, DGa, DGb, DGc, and DGd), whose recruitment is controlled by direct modulation of cell excitability (see Figure 1). In the biological DG, there can be some minimal overlap among contextual ensembles, and new ensembles can constantly be created due to neurogenesis (Luu et al., 2012); so our model is only a very simple test of the concept. In particular, the excitability of context-inappropriate DG cells is temporarily suppressed by raising the maximal conductance of their lateral inhibitory channels (namely, $\bar{g_{i}}$ in Equation 2); see Table 1. When PFC is inactivated, there is a loss of this contextual inhibitory control over the DG. In other words, the excitability of all DG cells remains at normal levels without any top-down relative bias for a particular contextual ensemble.

**Table 1:** Model parameters.

**Parameter**	**Equation**	**LEC (II/III, V/VI)**	**DG**	**CA3,CA1**
$\bar{g_{l}}$	(2)	0.1	0.1	0.1
$\bar{g_{e}}$	(2)	1	1	1
$\bar{g_{i}}$	(2)	1	1 or 5	1
(Υ, θ)	(4)	(100, 0.5)	(100, 0.5)	(100, 0.5)
(*E*_*l*_, *E*_*e*_, *E*_*i*_)	(2)	(0.3, 1, 0.25)	(0.3, 1, 0.25)	(0.3, 1, 0.25)
*V*_*rest*_	(2)	0.3	0.3	0.3
*q*	(5)	0.25	0.25	0.25
*kinkWTA*	(5)	25%	1%	2.5%

*Values for various parameters in each sub-region within our entorhinal-hippocampal model that were used to simulate the experimental data related to the effects of PFC inactivation on memory encoding and recall behavior. Parameter values for the connections among various sub-regions are provided in Table 2. Note that all sub-regions share the same parameter values, except for the level of sparseness in activity (namely, k in Equation 5). Consistent with data (Jung and McNaughton, 1993; Chawla et al., 2005), DG is the sparsest compared to CA3 and CA1 in the hippocampus (archicortex), which are still less sparse relative to the superficial and deep layers of LEC (neocortex). And note $\bar{g_{i}}$ is set to 1 for the DG ensemble assigned to a current context and set to 5 for other DG ensembles assigned to other contexts*.

Context *A* is used to simulate the data for Experiments 1A and 1B in Peters et al. (2013), while contexts *A* and *B* are both employed for the other experiments. We devised a simple scheme to emulate the innumerable contexts the rats may have experienced in their lifetimes, for which they have formed memories that could potentially interfere with encoding stimuli in these contexts. For this purpose, two odor discrimination lists with high interference (overlap of one odor in each problem presented in the contexts at hand) were designed. Simulated rats acquired these lists for five blocks each in the other two contexts *C* and *D* before the experimental manipulations. See section 3.1 for how each simulated rat was instantiated.

For all experiments, the retrieval performance *P* (%) in response to each discrimination problem was assessed by using a similarity metric based on the root mean squared error (RMSE), *E*, between the correct pattern *R*_*targ*_ and the pattern *R*_*out*_ recalled in the third field of the deep layers of LEC (ECout), which represents the cortical read-out of the hippocampal recall process, as follows:

(1) $$P(\%)=min\left(50\%,100\times \frac{{\left(1-E\right)}^{10}}{0.5^{10}+{\left(1-E\right)}^{10}}\right),$$

where *E* is in the range 0−1. Note Equation 1 ensures that chance performance is 50%, as the rats have to choose between two odors for each discrimination problem. We now briefly describe how each of the experiments was simulated.

Navawongse and Eichenbaum (2013): Simulated rats (*N* = 10) learned a list of odor discrimination memories ({*X*_*i*_, *Y*_*i*_} → *X*_*i*_/*Y*_*i*_, i = 1…8) in context *A* across several blocks until criterion performance was achieved (i.e., 90% accuracy in two consecutive blocks) and then learned a list of conflicting associations ({*X*_*i*_, *Y*_*i*_} → *Y*_*i*_/*X*_*i*_, i = 1…8) in context *B* to criterion. This training phase was followed by blocks of test trials ({*X*_*i*_, *Y*_*i*_} → ?, i = 1…8) in each context to assess memory retrieval performance with saline or muscimol injections into PFC. As mentioned above, we simulated the muscimol condition by allowing all DG ensembles to potentially become active (with a lower $\bar{g_{i}}$), whereas we simulated the saline (control) condition by selectively increasing $\bar{g_{i}}$ for all DG ensembles except the one that is pertinent to the current context. We also simulated prior experiences that could potentially interfere with the new memories of the experiment by pre-training the rats on interfering lists of discrimination problems in contexts *C* and *D* for 10 blocks each (*C*:{*X*_*i*_, *Z*_*i*_} → *X*_*i*_/*Z*_*i*_, *i* = 1…8); (*D*:{*Z*_*i*_, *Y*_*i*_} → *Z*_*i*_/*Y*_*i*_, i = 1…8).

Peters et al. (2013) – Experiment 1A: Simulated rats (*N* = 10 for normal PFC [control]; *N* = 10 for inactivated PFC [muscimol]) learned a list of odor discrimination memories ({*X*_*i*_, *Y*_*i*_} → *X*_*i*_/*Y*_*i*_, *i* = 1…8) in context *A* across several blocks until criterion performance was achieved (i.e., 90% accuracy in two consecutive blocks). Each discrimination problem was presented once per block in random order. Muscimol rats had their PFC inactivated only during the first three training blocks. For each rat, once criterion was reached, four test blocks were conducted to assess memory retrieval performance. As in the experiment, PFC inactivation was applied to the first three test blocks for the simulated control rats only. We simulated prior experiences that could potentially interfere with the new memories of the experiment by pre-training the rats on interfering lists of discrimination problems in contexts *C* and *D* for 10 blocks each (*C*:{*X*_*i*_, *Z*_*i*_} → *X*_*i*_/*Z*_*i*_, *i* = 1…8); (*D*:{*Z*_*i*_, *Y*_*i*_} → *Z*_*i*_/*Y*_*i*_, *i* = 1…8).

Peters et al. (2013) – Experiment 1B: Simulated rats (*N* = 10 for normal PFC [control]; *N* = 10 for inactivated PFC [muscimol]) were trained to learn one odor discrimination problem at a time to criterion ({*X*_*i*_, *Y*_*i*_} → *X*_*i*_/*Y*_*i*_, *i* = 1…10), unlike the concurrent acquisition in Experiment 1A. The relative order for acquiring the memories was chosen randomly for each rat. For muscimol rats, all training trials occurred under PFC inactivation. We also simulated prior experiences that could potentially interfere with the new memories of the experiment by pre-training the rats on interfering lists of discrimination problems in contexts *C* and *D* for 10 blocks each (*C*:{*X*_*i*_, *Z*_*i*_} → *X*_*i*_/*Z*_*i*_, *i* = 1…8); (*D*:{*Z*_*i*_, *Y*_*i*_} → *Z*_*i*_/*Y*_*i*_, *i* = 1…8).

Peters et al. (2013) – Experiment 2: Simulated rats (*N* = 40) were first trained in context *A* to learn to criterion the same list of discrimination problems that was used for Experiment 1A. They were then trained on a new list of memories for five blocks. As mentioned earlier, these two lists conflicted as follows: for each context *A* memory (say, {*X*_1_, *Y*_1_} → *Y*_1_), one of the two odor cues was randomly replaced by a new cue (say, *W*_1_ instead of *Y*_1_) and the reward prediction of the remaining cue from context *A* (i.e., *X*_1_) was reversed leading to {*X*_1_, *W*_1_} → *X*_1_. Rats were exposed to List 2 either in the same context *A* (*N* = 10) or a different context *B* (*N* = 10), with either a normal (*N* = 10) or an inactivated (*N* = 10) PFC. In other words, this experiment employed a 2x2 design with the following subsets of rats: control – different context, control – same context, muscimol – different context, and muscimol – same context. As in Experiment 1A, muscimol rats had their PFC inactivated only for the first three blocks to learn List 2. We also simulated prior experiences that could potentially interfere with the new memories of the experiment by pre-training the rats on interfering lists of discrimination problems in contexts *C* and *D* for 10 blocks each (*C*:{*X*_*i*_, *Z*_*i*_} → *X*_*i*_/*Z*_*i*_, *i* = 1…8); (*D*:{*Z*_*i*_, *Y*_*i*_} → *Z*_*i*_/*Y*_*i*_, *i* = 1…8).

Peters et al. (2013) – Experiment 3: Simulated rats (*N* = 10 for normal PFC [control]; *N* = 10 for inactivated PFC [muscimol]) were first trained in context *A* for five blocks with the same list of discrimination problems used in Experiment 1A, and then trained on the second, conflicting list from Experiment 2 for five blocks in the same context *A*. PFC inactivation for the muscimol group occurred only during the first three training blocks for List 1. We also simulated prior experiences that could potentially interfere with the new memories of the experiment by pre-training the rats on interfering lists of discrimination problems in contexts *C* and *D* for 10 blocks each (*C*:{*X*_*i*_, *Z*_*i*_} → *X*_*i*_/*Z*_*i*_, *i* = 1…8); (*D*:{*Z*_*i*_, *Y*_*i*_} → *Z*_*i*_/*Y*_*i*_, *i* = 1…8).

### 3.1. Model equations

We now provide structural and functional details of our model, including values for the various parameters. The model simulates associative memory formation and recall using dynamic sparse ensemble codes and activity-dependent synaptic plasticity in the recurrent connections within the hippocampus. Simulations were performed in the *emergent* neural network simulator (O'Reilly and Munakata, 2000; Aisa et al., 2008), whose underlying equations are described in detail below. Following Ketz et al. (2013), we simulated a cycle of three different phases of hippocampal processing during each trial of memory formation; namely, first and second halves of the *minus* phase, followed by the *plus* phase. In the first half of the *minus* phase, the trisynaptic pathway is suppressed, inhibiting the CA3-based recall of previous memories. But the monosynaptic pathway is still active, which enables the activity pattern in CA1 (decoupled from CA3) to be learned as an auto-encoded representation of the EC inputs. In the second half of the *minus* phase, the monosynaptic pathway is suppressed in favor of the trisynaptic pathway. Memory recall thus coincides with the second half of the *minus* phase, when the system expectation is registered in ECout as a result of pattern completion in CA3. In this way, CA1 activity is controlled by different input streams in the different *minus* phases. The *emergent* framework also posits a *plus* phase that is similar to the first half of the *minus* phase but with ECout clamped to the incoming cortical patterns representing the memory association (in the streams for the input cues and the rewarded cue). The patterns in the cortical areas upstream to ECin are clamped during all phases in each trial.

During memory acquisition, the synaptic weights in the network change in an activity-dependent manner at the end of the *plus* phase using a combination of Hebbian and error-driven learning. For Hebbian learning, the weight changes depend on just the *plus* phase activities. For error-driving learning, the weight changes for connections depend on activities in the *plus* phase and the corresponding *minus* phase. In particular, synaptic weight changes in the monosynaptic pathway (ECin → CA1, CA1 → ECout, ECout → ECin) depend on activities in the *plus* phase and the first half of the *minus* phase. Of particular importance, during memory acquisition, the CA3 → CA1 connections are adapted such that the pattern-completing CA3 activity can reproduce the auto-encoded representation in CA1 during subsequent cue-based recall. There are now more biologically plausible implementations of error-driven learning for training multi-layered recurrent neural networks (Liao et al., 2016; Lillicrap et al., 2016; Hassabis et al., 2017; Scellier and Bengio, 2017; Whittington and Bogacz, 2017) without requiring matching feedforward and feedback connectivity and non-local information for updating weights.

The model equations are described next, and values for various model parameters are listed in Tables 1, 2. The membrane potential *V*_*m*_ of a given model cell is defined by Equation 2 that obeys shunting dynamics within a recurrent competitive network:

(2) $$\begin{array}{l} \frac{dV_{m}(t)}{dt}=\tau [{\bar{g}}_{l}(E_{l}-V_{m}(t))+g_{e}(t){\bar{g}}_{e}(E_{e}-V_{m}(t))\\ \;+g_{i}(t){\bar{g}}_{i}(E_{i}-V_{m}(t))], \end{array}$$

where τ scales the rate of the cell's temporal integration; $\bar{g_{l}}$ is the constant conductance of the leak *Cl*^−^ channel; *E*_*l*_ is the reversal potential of the leak channel; $\bar{g_{e}}$ is the maximal conductance of each excitatory channel and *E*_*e*_ is the corresponding reversal potential; $\bar{g_{i}}$ is the maximal conductance of each inhibitory channel and *E*_*i*_ is the corresponding reversal potential; *g*_*e*_ is the net synaptic weight of excitatory channels controlled by cells either within the network or from other networks; and *g*_*i*_ is the net synaptic weight of inhibitory channels controlled by cells within the network. Equation 2 was numerically integrated using Euler's forward method with a fixed time step Δ*t* = 1 ms. The net excitatory synaptic weight *g*_*e*_ is defined as follows:

(3) $$\begin{array}{r} g_{e}\left(t\right)=\underset{j}{\sum }\underset{h}{\sum }\left(\frac{r_{j}}{\underset{k}{\sum }r_{k}}\right)a_{j}{w_{h}}^{j}, \end{array}$$

where ${w_{h}}^{j}$ is the synaptic weight of the excitatory projection from the *h*^*th*^ cell in the *jth* network; *r*_*j*_ is the relative weight for the *jth* network that is normalized by net relative weight for all incoming networks; and *a*_*j*_ is the absolute scaling parameter for the projections from the *jth* network. The output activity *y*(*t*) of the cell is computed using an activation function that operates on the membrane potential as follows:

(4) $$\begin{array}{r} y\left(t\right)=\frac{\chi }{1+\chi },\text{with}\;\chi =\Upsilon {\left[V_{m}\left(t\right)-\theta \right]}_{+}, \end{array}$$

where Υ is a scaling parameter; θ is the activity threshold on the membrane potential; and [ ]_+_ denotes the half-wave rectifier function. The net inhibitory synaptic weight *g*_*i*_ is a constant for all cells within the network such that only a given proportion of the cells (say, the top *k*) exhibit non-zero activities at any moment, and is defined as follows:

(5) $$\begin{array}{r} g_{\theta }^{k}=\frac{g_{e}\bar{g_{e}}\left(E_{e}-\theta \right)+\bar{g_{l}}\left(E_{l}-\theta \right)}{\theta -E_{i}},\text{and}g_{i}\left(t\right)=g_{\theta }^{k+1}+q\left(g_{\theta }^{k}-g_{\theta }^{k+1}\right), \end{array}$$

where 0 < *q* < 1, $g_{\theta }^{k}$ is the threshold inhibition for the cell with the *k*^*th*^ most activity in the network. These equations to determine *g*_*i*_, thus, implement *k*-Winner Take All (kWTA) inhibition leading to distributed representations with variable sparsities (O'Reilly and Munakata, 2000). They provide a simple computational approximation to the function of feedback inhibitory interneurons in real neural networks.

**Table 2:** Model connectivity.

**Projections between brain regions**	**Connectivity**	***k*_*hebb*_ (Equation 6)**	**Weight scale (absolute, relative)**
LEC II/III → DG (perforant path)	*p* = 25%	1	(1, 1)
LEC II/III → CA3 (perforant path)	*p* = 25%	1	(1, 1)
LEC II/III → CA1 (perforant path)	*p* = 25%	0.05	(1, 1)
DG → CA3 (mossy fibers)	*p* = 4%	1	(10, 3.5)
CA3 → CA1 (Schaffer collaterals)	full	1	(5, 1)
CA1 → LEC VI/VI (recurrent collaterals)	full	0.05	(1, 2)
LEC VI/VI → LEC II/III (intracortical)	one-to-one	0.05	(1, 1)

*Details of the connections among various sub-regions within our entorhinal-hippocampal model, including the type of connectivity [namely, one-to-one, full (each-to-all), random with probability], the proportion of Hebbian learning compared to error-driven learning (0 ≤ k_hebb_ ≤ 1 in Equation 6), and the values for absolute scaling parameter and relative weight (see a_j_ and r_j_ in Equation 3). Parameter ϵ, which scales the rate of learning, is set to 0.01 for all connections*.

All membrane potentials are initialized to *V*_*rest*_ at the start of each trial. But model CA1 cells are reset to *V*_*rest*_ at the start of each of the three theta phases in every trial. Additionally, cell activities in the deep layers of LEC (namely, ECout) are clamped during the *plus* phase (in the encoding trials) to the incoming pattern in the high-level cortical area upstream to LEC. In every encoding trial the three phases proceed in sequence as follows, each with a fixed number of time steps to allow for the activities to settle: first half of *minus* phase (30 steps) → second half of *minus* phase (30 steps) → *plus* phase (30 steps).

The adaptive weights, $w_{h}^{j}$, of the excitatory synaptic connection from the *h*^*th*^ cell in the *jth* network are updated at the end of *plus* phase in each encoding trial using a combination of Hebbian learning and error-driven learning (O'Reilly and Munakata, 2000):

(6) $$\begin{array}{r} \Delta w_{h}^{j}=\epsilon \left[k_{hebb}\left(\Delta w_{hebb}\right)+\left(1-k_{hebb}\right)\left(\Delta w_{err}\right)\right], \end{array}$$

where 0 ≤ *k*_*hebb*_ ≤ 1, ϵ scales the rate of learning; and *k*_*hebb*_ is a parameter that determines the proportion of Hebbian learning compared to error-driven learning in the mixture. The Hebbian (Δ*w*_*hebb*_) and error-driven (Δ*w*_*err*_) weight updates are defined as follows:

(7) $$\begin{array}{r} \Delta w_{hebb}=y^{+}\left(x_{h,j}^{+}-w_{h}^{j}\right), \end{array}$$

(8) $$\begin{array}{r} \Delta w_{err}=\left(x_{h,j}^{+}y^{+}\right)-\left(x_{h,j}^{-}y^{-}\right), \end{array}$$

where *x*_*h, j*_ is the activity of the projecting cell, and the superscripts + and – correspond to activities at the end of the *plus* phase and the applicable *minus* phase, respectively. Equation 7 is a variant of Hebbian learning that prevents the weights from growing without bounds (Grossberg, 1976; Rumelhart and Zipser, 1986; O'Reilly and Munakata, 2000). It ensures the selective strengthening of projections from those input cells that are consistently co-active with the post-synaptic cell during the *plus* phase. Equation 8, which is equivalent to the contrastive Hebbian learning rule (Hinton, 1990), ensures gradual matching between the activities at the end of *minus* (expectation) and *plus* (output) phases. The error-driven weight updates and the weights themselves are subject to soft bounding between the limits of 0 and 1. The weights of all present projections for each simulated rat are initialized at the beginning of experience by random sampling from a uniform distribution with mean 0.5 and variance 0.25; see Table 2 for probabilities related to sparse connectivity in the perforant path and mossy fiber projections.

## 4. Results and explanation

This section presents the simulation results of our model that explain the data from the odor discrimination experiments (Navawongse and Eichenbaum, 2013; Peters et al., 2013), which reveal the top-down modulation of context-based memory associations in the hippocampus and highlight the distributed nature of memory processes. With independent pools of DG cells that are facilitated one at a time for a given context under normal operation, our model can successfully retrieve context-appropriate memories and correctly discriminate in response to various pairs of odor cues. Even if the same stimulus is experienced in different contexts (say, odor *X*_1_), varied and potentially conflicting associations can nevertheless be learned (e.g., *X*_1_ ↔ *Y*_1_ in context *A*; *X*_1_ ↔ *Z*_1_ in context *B*); see **Figure 6** for an illustrated explanation. This is possible despite the direct perforant path projections from the input layers of LEC (ECin) to CA3 and CA1. Our model is capable of forming and recalling contextual memories, because not only are the different contexts distinguished within the DG, but also the mossy fiber projections from DG to CA3 are the strongest relative to other connections to CA3 cells (namely, EC → CA3 and CA3 → CA3); see Table 2.

### 4.1. How contextual bias affects memory

It is well accepted that DG plays a crucial role in mediating the important sub-function of “pattern separation” by virtue of sparse connectivity in its perforant path projections from EC, its much larger size compared to the input EC layers, and its sparse distributed activity (Marr, 1971; Jung and McNaughton, 1993; Treves and Rolls, 1994; McClelland et al., 1995). There is a variety of evidence that the DG helps to distinguish similar memories experienced in different contexts. The degree of overlap among sets of DG cells that get activated for various memories within two different contexts is just 1%, which is much less than the 30% overlap in CA1 (Thompson and Best, 1989; Ramirez et al., 2013). Moreover, the selection of a new ensemble of DG cells can be triggered by exposure to a new environment or change in the behavioral task (Chawla et al., 2005; Leutgeb et al., 2007; Satvat et al., 2011; Schmidt et al., 2012; Deng et al., 2013).

With its random network comprising extensive recurrent collaterals (Amaral and Witter, 1989), CA3 underlies the crucial sub-functions of “auto-association” during encoding and “pattern completion” during cue-triggered retrieval (Marr, 1971; Treves and Rolls, 1994). Recurrent connections among CA3 cells that are simultaneously activated by a current event are selectively strengthened in an activity-dependent manner, which thereby subsequently support pattern completion in response to a partial cue (McNaughton and Morris, 1987; Nakazawa et al., 2002). As noted above, DG projects to CA3 with extremely sparse but potent mossy fibers (Henze et al., 1997) contributing to auto-association and pattern completion to minimize interference from similar memories (Leutgeb et al., 2007). We propose that all these properties work in conjunction with, and are further enhanced, by the additional contextual recruitment of DG cells; see also Deng et al. (2013). In other words, the mossy fibers from DG to CA3 bias the selection of the appropriate attractor state within the CA3 recurrent network in response to cues shared between multiple familiar contexts.

**Figure 3:**
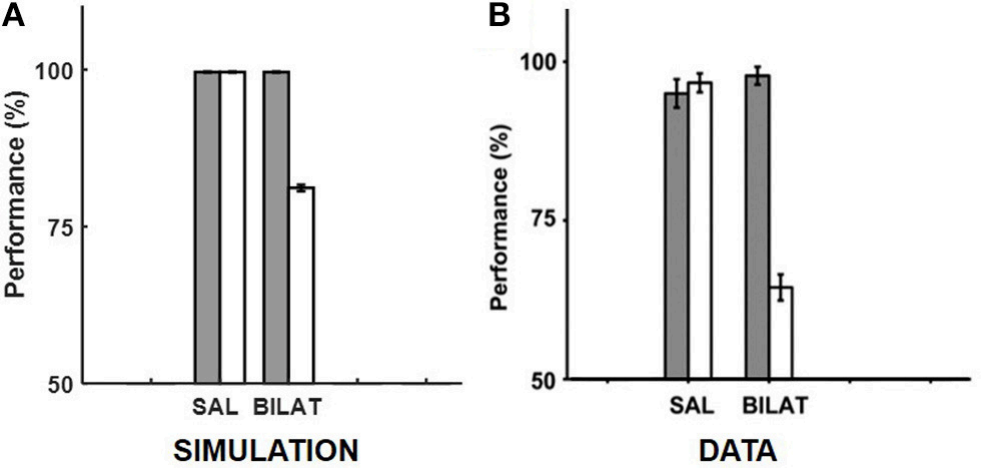
Comparison of our model with the rat experiment of Navawongse and Eichenbaum (2013). **(A,B)** provide our model simulation results and the memory retrieval performance data from Navawongse and Eichenbaum (2013), respectively, with (muscimol) and without (saline) PFC inactivation in the context-guided object association task. The gray bars correspond to baseline performances for retrieving conflicting memories that were acquired in two different contexts, and the white bars correspond to performances under either normal PFC operation with saline (SAL) or bilateral PFC inactivation with muscimol (BILAT). The error bars correspond to standard error of mean. Panel **(B)** is reprinted with permission from Navawongse and Eichenbaum (2013).

**Figure 4:**
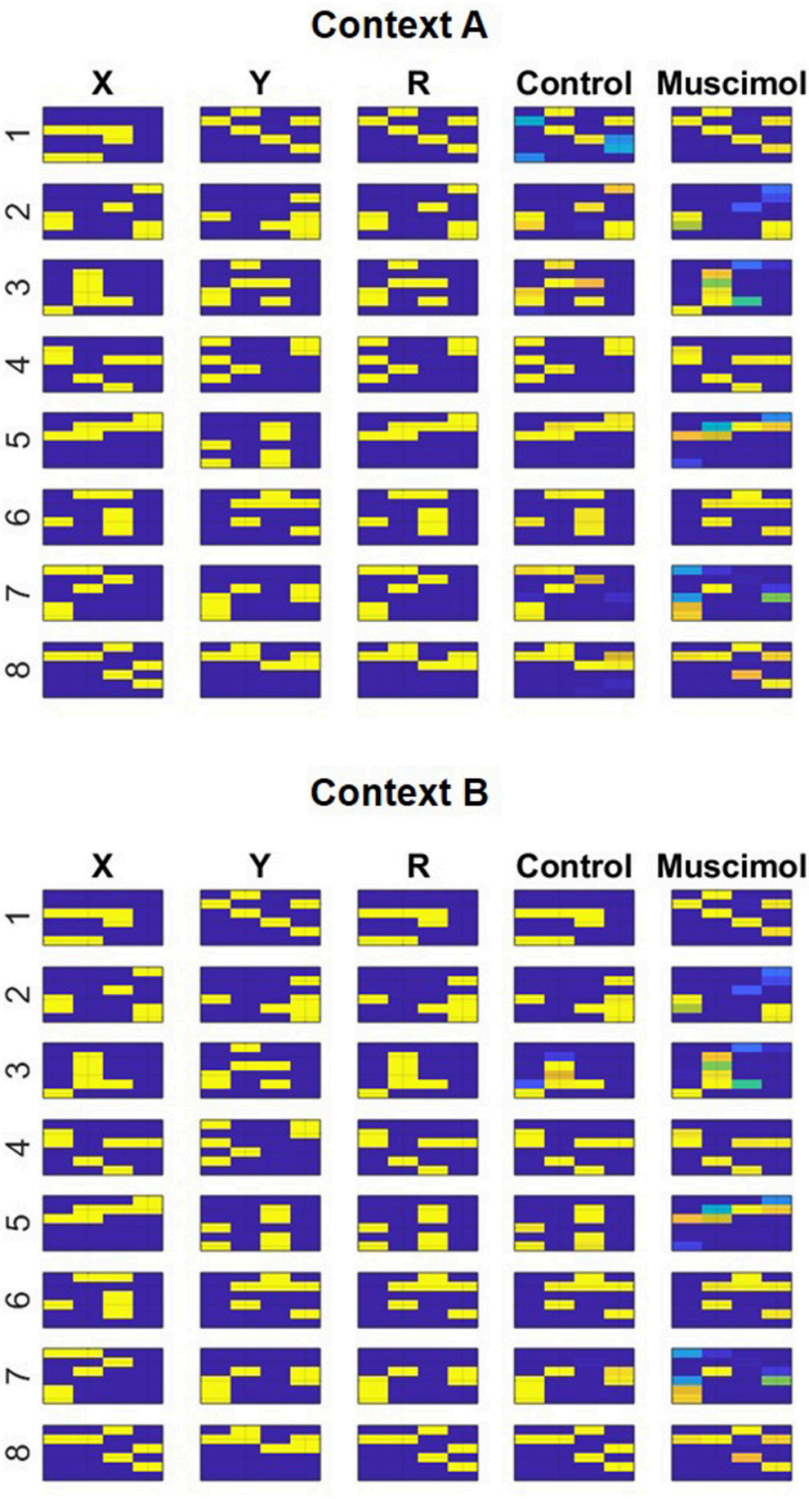
Modeling context-based retrieval with and without PFC inactivation in the rat experiment of Navawongse and Eichenbaum (2013). Following the learning of two conflicting lists of odor discrimination memories in different contexts (*A* and *B*), the control condition faithfully recalls the correct odor (*R*) for each pair ({*X*_*i*_, *Y*_*i*_}, *i* = 1…8) based on the context; see the match between columns titled “R” and “Control.” However, the muscimol condition reduces overall accuracy by recalling the incorrect odor for a few pairs of memories; for example, see the divergent recalls in the control and muscimol conditions for {*X*_6_, *Y*_6_} in context *A*. Note the activation patterns in columns titled “X,” “Y,” and “R” correspond to incoming high-level neural codes upstream of the superficial layers of LEC (ECin) during training. And the activation patterns in columns titled “Control” and “Muscimol” correspond to activities recalled for *R*_*i*_ in the third field of the deeper layers of LEC (ECout) at the end of the second half of the *minus* phase in response to inputs {*X*_*i*_, *Y*_*i*_} in columns titled “X” and “Y” during testing.

Given the specifics of the intra-hippocampal connectivity (see Figure 1 and Table 2), context-specific subsets could also be triggered in CA3 and CA1 downstream from DG. However, CA3 and CA1 would exhibit an appreciable overlap among cells participating in different contexts (Thompson and Best, 1989; Ramirez et al., 2013) owing to their smaller sizes and more densely distributed activities compared to DG. We hypothesize that the contextual mossy fibers from DG to CA3 can bias the selection of the appropriate attractor state within the CA3 recurrent network in response to cues shared among multiple familiar contexts. As in the experiment of Navawongse and Eichenbaum (2013)), our simulated rats learned to perform the contextual odor discrimination task almost perfectly. And they exhibited highly impaired performance when their PFC was inactivated [*t*_(9)_ = 33.52, *p* = 0; see Figure 3]. The ability to learn one or more memory associations in the same or different contexts for subsequent robust behavioral recall indicates the presence of stable attractor states in the CA3 recurrent network. Figure 4 shows the activity patterns recalled by a representative simulated rat for the correct choice in response to each pair of odors in both contexts with and without PFC inactivation during the test blocks. The Control column shows the recalled patterns for normal PFC operation, which faithfully match the corresponding trained *R* patterns. The Muscimol column shows the recalled patterns when PFC is inactivated, and it appears that the recalled patterns are a random choice between the corresponding patterns for contexts *A* and *B*, and not a mixture of the two contexts.

### 4.2. Concurrent and blocked acquisition of memories

**Figure 5:**
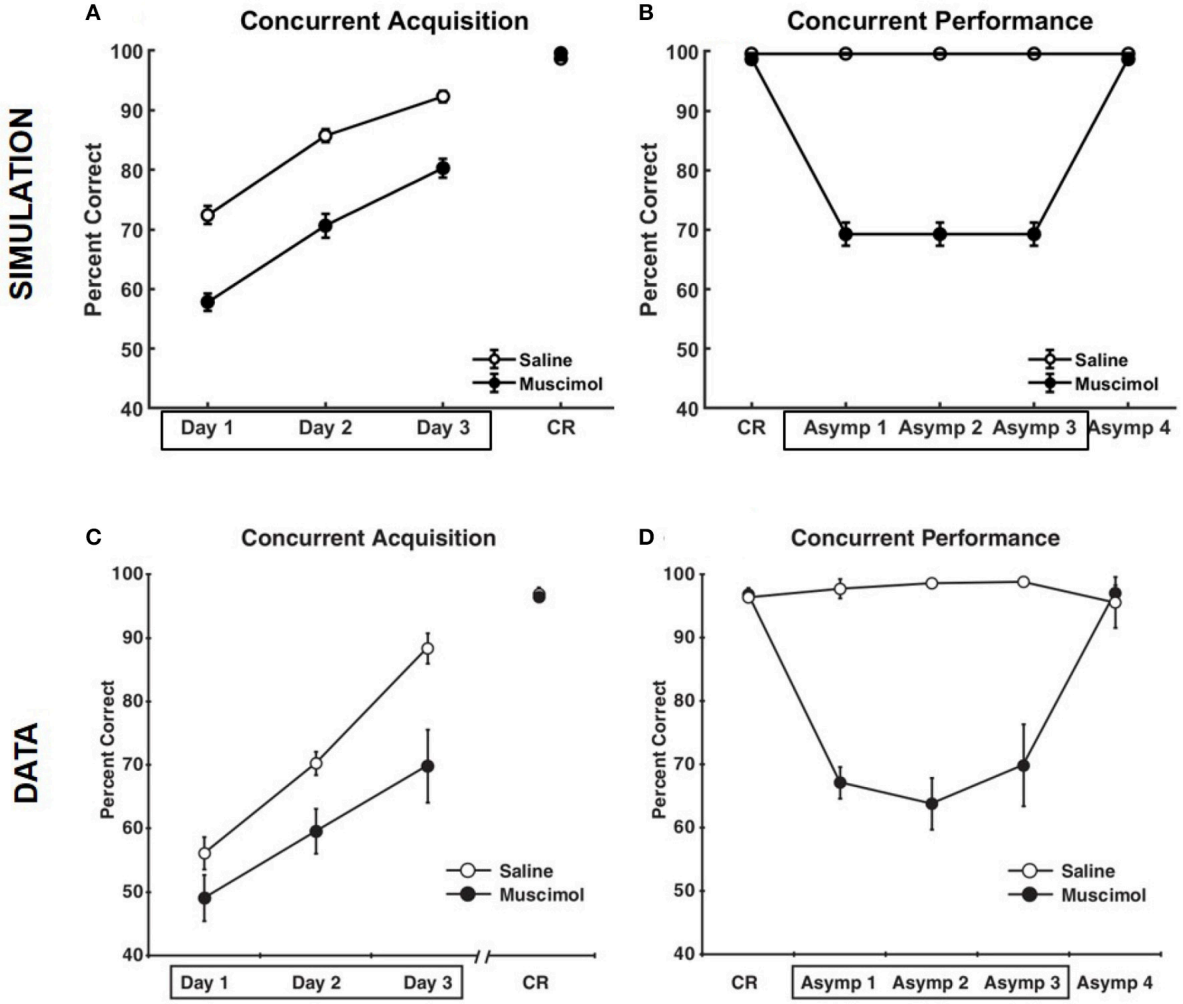
Comparison of our model with rat experiments 1A of Peters et al. (2013). Results of our model simulation are in the top row **(A,B)**, and the data from Experiment 1A of Peters et al. (2013) are in the bottom row **(C,D)**, which are related to the effects of PFC inactivation on concurrent acquisition **(A,C)** and concurrent performance **(B,D)** of multiple odor discrimination memories. The muscimol condition corresponds to PFC inactivation during the first three training blocks (depicted by the box in **A,C**), and the saline condition is the control. CR on the x-axis in all panels refers to the training block in which rats reached criterion performance. Control rats with saline injection during acquisition received muscimol infusion during the three test blocks following acquisition to criterion (depicted by the box in **B,D**), but not the muscimol rats. The error bars correspond to standard error of mean. Panels **(C,D)** are reprinted with permission from Peters et al. (2013).

Figure 5 compares the results of our model simulation with the data from Experiment 1A of Peters et al. (2013), which shows that PFC inactivation can affect the ability to discriminate various pairs of odors even if they have only been presented in a single context. The model simulated 10 control rats and 10 muscimol rats, with each simulated rat being presented with a sequence of eight discrimination problems in random order on each epoch until a criterion level of performance was reached. The performance data of the simulated rats from the first three training blocks was analyzed using a two-way repeated measures ANOVA with inactivation condition and training block as factors. Both simulated control and muscimol rats showed evidence of learning, with a significant main effect of training block [*F*_(2, 54)_ = 94.49, *p* = 0], as in the data (see Figures 5A,C). And similar to the data, simulated muscimol rats learned the memory associations less accurately than simulated control rats initially, with a significant main effect of inactivation condition [*F*_(1, 54)_ = 119.64, *p* = 0]. Also matching the data, there was no interaction between PFC inactivation and training block in our model [*F*_(2, 54)_ = 0.56, *p* = 0.5737]. The performance data of the simulated rats from the first three test blocks, after concurrent acquisition to criterion performance, was also analyzed using a two-way repeated measures ANOVA with inactivation condition and training block as factors. As in the data, muscimol infusion for the simulated control rats severely impaired their ability to recall well-formed memories, with a significant main effect of inactivation condition [*F*_(1, 54)_ = 654.09, *p* = 0; see Figures 5B,D]. Finally, when PFC inactivation ceased in these simulated control rats for the last test block, retrieval behavior returned to about the same level as the simulated muscimol rats, as seen in the data.

**Figure 6:**
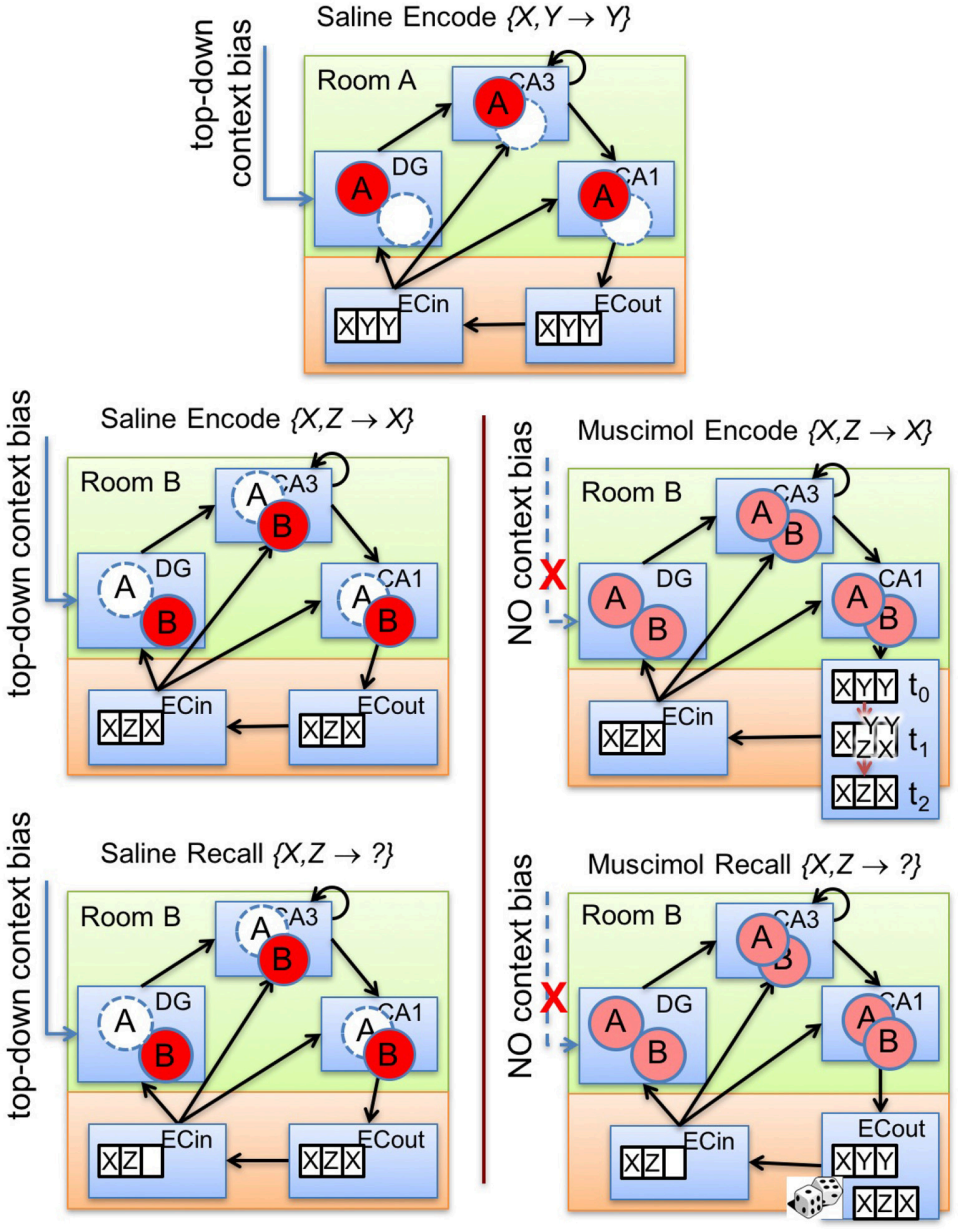
Illustration of our theory. Top-down bias on DG affects memory encoding and recall by controlling the activation of memory traces within the hippocampal subfields (namely, DG, CA3, CA1). The top panel exemplifies prior experience where an associative memory {*X, Y*} → *Y* is encoded in room *A* with top-down bias from PFC. The other panels depict the encoding (second row) and retrieval (third row) for a conflicting associative memory {*X, Z*} → *X* in room *B* with (lower left panels) and without (lower right panels) the context bias. Note that normal PFC operation allows only context-appropriate memory traces to become active. With PFC inactivation, the learning of the conflicting association {*X, Z*} → *X* in room *B* is slowed because of the interfering memory traces triggered by concurrent activation of granule cells in the two DG contextual ensembles in response to the common cue *X*. If the two memories {*X, Y*} → *Y* and {*X, Z*} → *X* were acquired in contexts *A* and *B*, respectively, under normal conditions, PFC inactivation during recall performance {*X, Z*} → ? in context *B* would also lead to concurrent activation of memory traces related to both associations in response to the common cue *X* in all hippocampal subfields starting with DG. Pattern completion processes in CA3 would then probabilistically converge to either memory engram in different trials, leading to impaired retrieval behavior.

Figure 6 illustrates our explanation for the impairment of memory encoding and retrieval that results from PFC inactivation. In the absence of inhibitory control by top-down contextual signals (see Figure 6, lower right panels), various sorts of context-inappropriate memory traces simultaneously emerge in the subfields of the hippocampus. In our model, the cause of these interfering signals is the indiscriminate activation of cells within the multiple contextual ensembles in DG to varying degrees in response to the current input. These DG traces promote the activation of their corresponding attractor states within the CA3 recurrent network via their previously tuned mossy fiber projections. The presence of multiple potential CA3 attractor states proactively conflicts with the auto-associative learning of the relevant activity pattern within CA3 during encoding. In particular, CA3 could randomly slip into one of the attractor states in each training trial. As a result, the trial-to-trial learning of CA3-to-CA1 Schaffer collaterals will be slower because of the lack of consistency in the CA3 activity pattern for the same discrimination problem across multiple acquisition trials while the PFC is inactivated. Moreover, the tuned Schaffer collaterals from CA3 cells to CA1 cells, and the tuned connections from CA1 cells to ECout cells, representing a previously encoded memory engram (to which CA3 converged) will also offer some inertia to the formation of the new memory. Thus, the consequent activation of conflicting memory traces in CA3 not only slows the learning of new contextual memories (see Figure 6, right panel in second row), but also impairs the cue-based retrieval of previously learned context-specific memories. Similar to encoding under PFC inactivation, during recall as well, there would be competition among multiple potential attractor states within CA3. Further, it is possible that the CA3 recurrent network may not converge to any previously learned memory engram owing to the fragmentary nature of the activated traces within various DG pools. In any case, the previously tuned CA3 → CA1 and CA1 → ECout projections will likely interfere with the generation of the correct recall pattern in the output layers of LEC (Figure 6, lower right).

**Figure 7:**
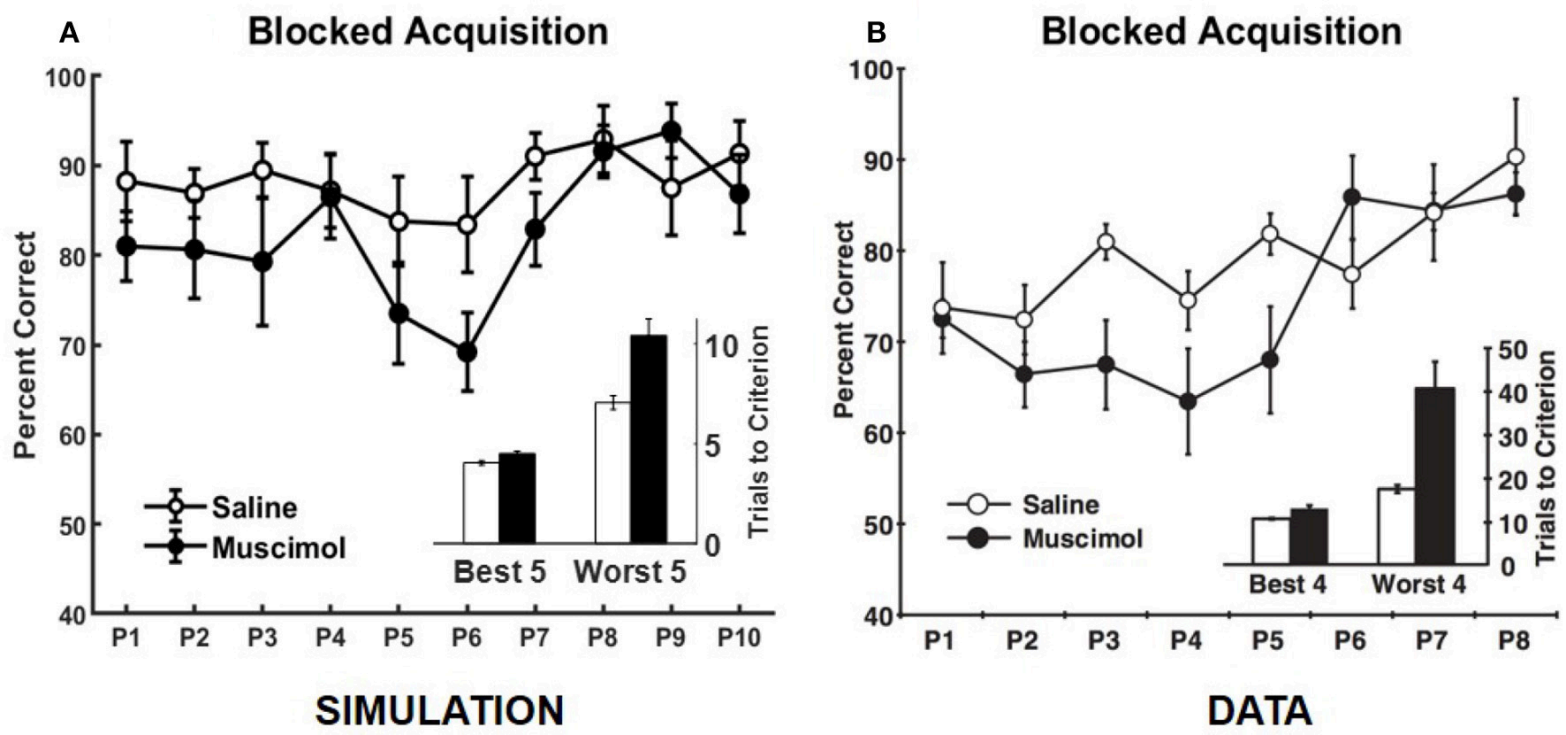
Comparison of our model with rat experiment 1B of Peters et al. (2013). Results of our model simulation are in **(A)**, and the data from Experiment 1B of Peters et al. (2013) are in **(B)**, which are related to the effects of PFC inactivation on the blocked acquisition of several memories one at a time to criterion. The muscimol condition corresponds to PFC inactivation, and the saline condition is the control. Overall performance for each discrimination problem (P#) is shown separately. The inset in either panel shows the number of trials to criterion for the saline and muscimol conditions with the discrimination problems divided into two halves based on learning difficulty. The error bars correspond to standard error of mean. Panel **(B)** is reprinted with permission from Peters et al. (2013).

Our model also simulates the data from Experiment 1B of Peters et al. (2013), in which rats learned each odor discrimination memory one at a time to criterion before training on another in the set (see Figure 7). As in the data, simulated muscimol rats were slower overall in acquiring the memories [trials to criterion difference: *t*_(18)_ = −3.85, *p* = 0.0012], and less accurate through their blocked acquisition [percent correct difference: *t*_(18)_ = 2.52, *p* = 0.022]. When the odor discrimination problems were separated into the best and worst halves according to the number of trials to criterion, the model shows a significant interaction between the discrimination difficulty and inactivation condition factors [*F*_(1, 36)_ = 21.03, *p* = 0.0051; two-way repeated measures ANOVA], as in the data. Peters et al. (2013) interpreted this data as implying that PFC is not critical when acquiring memories one at a time in a single context, but that PFC is essential when many discrimination problems are to be learned and remembered at the same time. In contrast, our simulations suggest that interference from older memories is potentially present even for blocked acquisition of single memories, and the strength of interference is not equal for all memories. In addition, the encoding process cannot be equal for all memories. It depends on the particular high-level cortical patterns that need to be encoded and the precise connectivity structure within the entorhinal-hippocampal system at the time of encoding. In other words, each discrimination problem does not have the same exact probability of being encoded. Thus, any untuned discrepancies between the experimental and simulated data, such as the differences in Day 1 performance between the saline and muscimol conditions for the concurrent acquisition of memories (see Figure 5) and in the overall number of trials to criterion for the blocked acquisition of memories (see Figure 7), are likely due to the particular neural codes chosen for the high-level cortical representation of the odor cues, the particular values chosen for the various parameters, and the particular initial entorhinal-hippocampal connectivity that was randomly sampled for each simulated rat, among others.

### 4.3. Acquisition of conflicting memories

**Figure 8:**
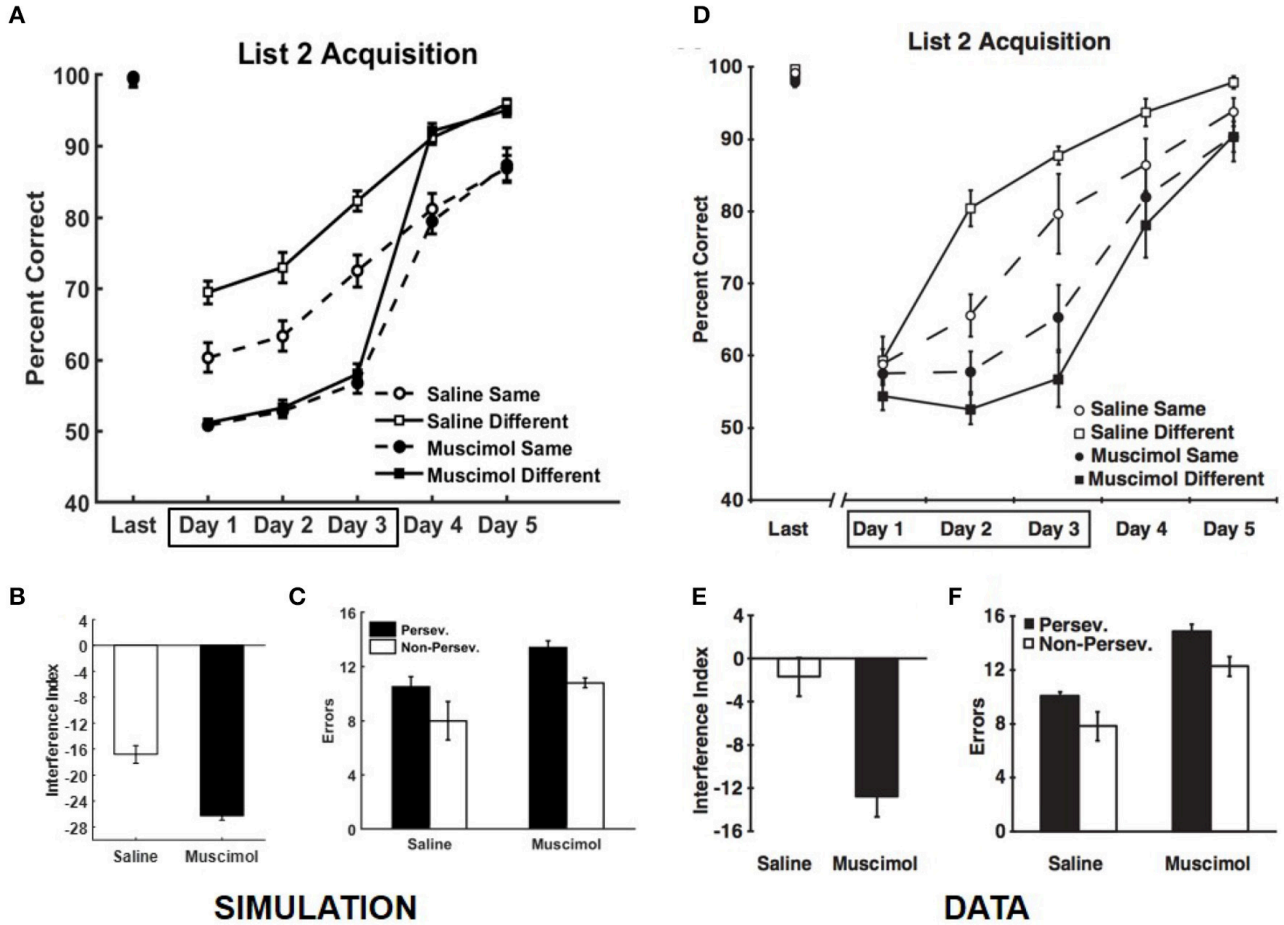
Comparison of our model with rat experiment 2 of Peters et al. (2013). Results of our model simulation are in **(A–C)**, and the data from Experiment 2 of Peters et al. (2013) are in **(D–F)**, related to the effects of PFC inactivation on the acquisition of List 2 memories, which conflict with List 1 memories, under various conditions. The muscimol condition corresponds to PFC inactivation during the first three blocks for List 2 acquisition (depicted by the box in **A,D**), and the saline condition is the control. Same and different conditions correspond to whether List 2 is acquired in the same or different context as List 1. **(B,E)** provide the amount of interference from List 1 on the acquisition of List 2 memories (called Interference Index) for the two PFC inactivation conditions. Panels **(C,F)** show the overall number of perseverative and non-preservative errors in the discriminative choices while acquiring List 2 memories. The error bars correspond to standard error of mean. Panels **(D–F)** are reprinted with permission from Peters et al. (2013).

Our simulations match the data from Experiment 2 of Peters et al. (2013), which is related to the effects of PFC inactivation on the dynamics of acquiring conflicting memories in the same and different contexts (see Figure 8). We simulate the data from control rats on the well-known effect of slower learning in a context where conflicting memories were previously formed (Barnes and Underwood, 1959). In this experiment, the rats were required to learn a new set of odor discrimination problems either within the same context or a different context, with and without PFC inactivation, where the new set conflicted with the set used in Experiment 1A. Here the memories of the two lists overlapped in one component, so that one odor in each *X*_1_, *Y*_1_ pair was retained and the reward prediction was reversed compared to the first set. For example, for the episode {*X*_1_, *Y*_1_} → *X*_1_ , if *X*_1_ were retained then the new event would be {*X*_1_, *Z*_1_} → *Z*_1_ ; whereas if *Y*_1_ were retained then the new event would be {*Z*_1_, *Y*_1_} → *Z*_1_ (*Z*_1_ is a new pattern, distinct from either *X*_1_ or *Y*_1_). The four different groups of simulated rats (PFC inactivation x context) first learned the list of memories employed in Experiment 1A (namely, List 1) and showed no difference in retrieval performance following acquisition to criterion [*F*_(3, 36)_ = 1.17, *p* = 0.33]. The performance data of the simulated rats from the first three training blocks for List 2 was analyzed using a two-way ANOVA with inactivation and context conditions as the between subject factors and training block as the within subject factor. Similar to the data, this analysis revealed a significant main effect of inactivation condition [*F*_(1, 110)_ = 303.76, *p* = 0] and a significant interaction between inactivation and context conditions [*F*_(1, 110)_ = 22.29, *p* = 0]; see Figures 8A,D. Simulated control rats exhibited a significant main effect of context condition [*F*_(1, 54)_ = 32.28, *p* = 0], while simulated muscimol rats did not differentiate between the same and different context conditions [*F*_(1, 54)_ = 0.62, *p* = 0.44] with respect to encoding the new memories from List 2, which interfered with older memories from List 1. Further, the interference from List 1 when learning List 2 memories was higher for simulated muscimol rats than simulated control rats [*t*_(18)_ = 5.99, *p* = 0; Figure 8B], as in the data shown in Figure 8E. This was quantified using an interference index metric, which is defined as the difference between the average percent correct for List 2 vs. that for List 1 in their first five training blocks (Peters et al., 2013).

The errors that occur during the learning of the conflicting List 2 memories are of two types: perseverative and non-perseverative. Consider an odor that was employed in both Lists 1 and 2 and was rewarded in List 1, but not in List 2 by design. If the rat makes an error by choosing it during List 2 acquisition, it would be a perseverative error (i.e., choosing the same odor despite the change from List 1 to List 2). Along the same lines, a non-perseverative error is one in which a new odor in a List 2 problem is chosen when it is paired with an unrewarded odor from List 1. The number of errors made by the simulated rats during the five training blocks of List 2 was analyzed by a two-way repeated measures ANOVA with inactivation condition and error type as factors. As in the data, there were more perseverative errors than non-perseverative errors [significant main effect of error type, *F*_(1, 36)_ = 7.79, *p* = 0.0084], and while the simulated muscimol rats made more errors than the simulated control rats [significant main effect of inactivation condition, *F*_(1, 36)_ = 9.73, *p* = 0.0036], the proportion of perseverative and non-perseverative errors was not significantly different between the simulated control and muscimol rats [no interaction between error type and inactivation condition, *F* = 0.16, *p* = 0.70; two-way ANOVA]; see Figures 8C,F.

When learning a List 2 problem in the same context, interference occurs because of the activation of conflicting memory traces (related to the corresponding List 1 problem) in each of the hippocampal subfields in general (and CA3 in particular). The projections from each CA3 engram corresponding to a List 1 associative memory to CA1, as well as auto-associative projections within CA3, need to overcome their prior tuning before new connections can be established to be able to correctly retrieve the List 2 memory. This impairment, which slows encoding, does not occur if List 2 memories are acquired in a new context; note that the “Saline Different” condition is uniformly better than “Saline Same” in Figure 8A, as the data shown in Figure 8D. When PFC is inactivated, there is even greater interference because the lack of top-down bias over DG allows innumerable traces within various contextual ensembles of DG cells that respond partially to the current association to be activated (see Figure 6). This includes the memory traces corresponding to List 1. Acquisition of List 2 memories was thus drastically affected by PFC inactivation, and also did not benefit from employing a different context. In the model, both perseverative and non-perseverative errors during List 2 learning occur because of interference from prior associations, or lack thereof, to the component of the memory engram signifying the rewarded odor. So, the relative proportion of perseverative and non-preservative errors did not significantly differ between the simulated control and muscimol rats.

### 4.4. Long-term effects of PFC inactivation

**Figure 9:**
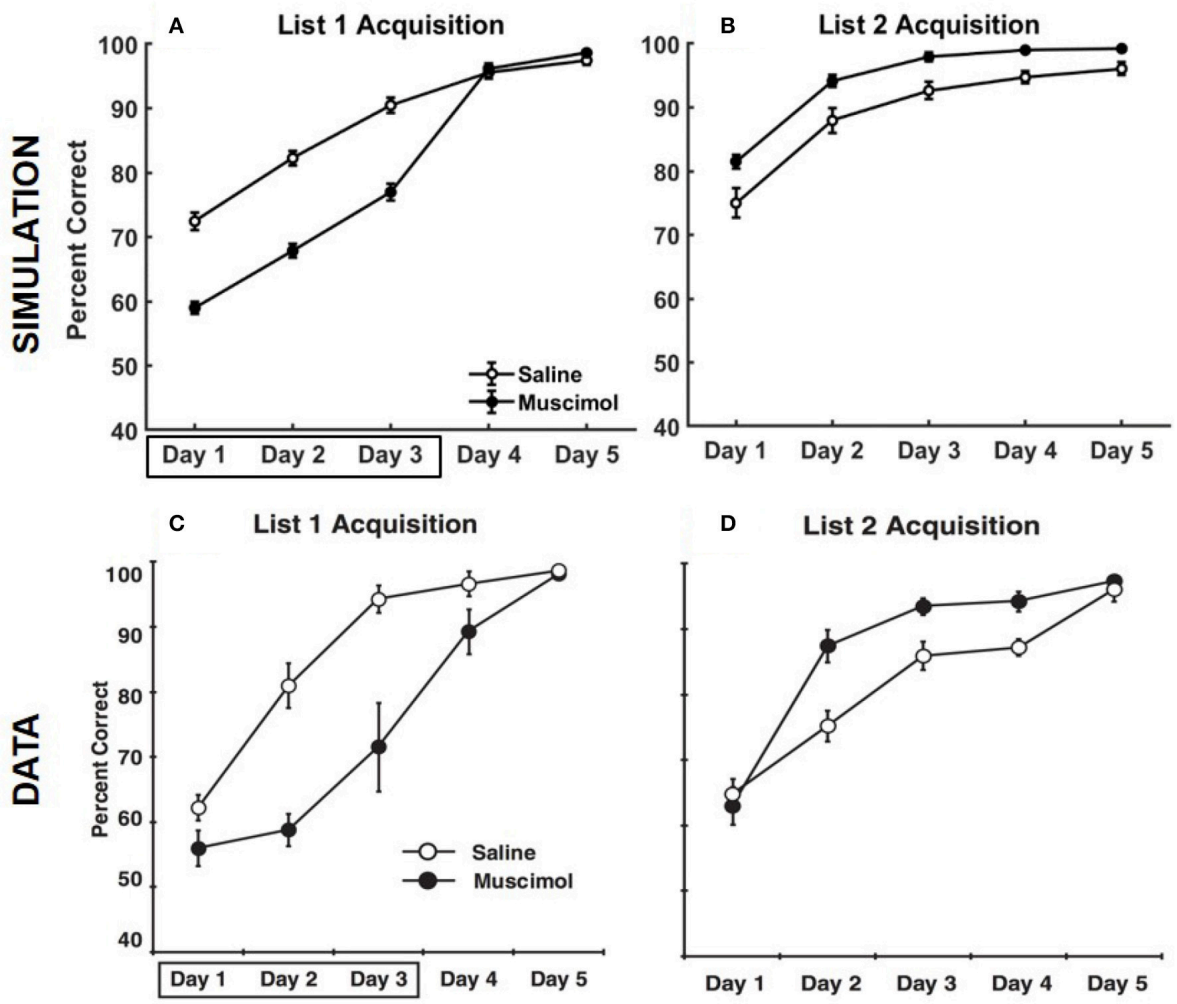
Comparison of our model with rat experiment 3 of Peters et al. (2013). Results of our model simulation are in the top row **(A,B)**, and the data from Experiment 3 of Peters et al. (2013) are in the bottom row **(C,D)**, which are related to the acquisition of List 2 memories (right column) following the encoding of conflicting List 1 memories (left column) in the same context with and without PFC inactivation. The muscimol condition corresponds to PFC inactivation during only the first three blocks of List 1 acquisition (depicted by the box in **A,C**), and the saline condition is the control. The error bars correspond to standard error of mean. Panels **(C,D)** are reprinted with permission from Peters et al. (2013).

Figure 9 provides the results of our model simulation of Experiment 3 of Peters et al. (2013), showing rats that learned the first set of odor memory associations with a deactivated PFC were surprisingly better at acquiring the second set of interfering memories in the same context under normal PFC operation. Similar to Experiment 1A, the performance data of the simulated rats from the first three training blocks of List 1 was analyzed using a two-way repeated measures ANOVA with inactivation condition and training block as factors. As in the data, simulated muscimol rats were initially less accurate in learning List 1 memories compared to simulated control rats [significant main effect of inactivation condition, *F*_(1, 54)_ = 186.28, *p* = 0]. Also, there was a significant main effect of training block [*F*_(2, 54)_ = 105.97, *p* = 0], but no interaction between inactivation condition and training block [*F*_(2, 54)_ = 0.1, *p* = 0.91]. Though the simulated muscimol rats were initially impaired, the performance was not different between the saline and muscimol conditions by the fifth training block [*t*_(18)_ = −1.41, *p* = 0.17]. Next, the performance data of the simulated rats from the five training blocks of List 2 was analyzed using a two-way repeated measures ANOVA with inactivation condition and training block as factors. Note that muscimol rats had their PFC inactivated only during the learning of List 1 for the first three blocks, and not during the learning of List 2. As in the data, simulated muscimol rats were surprisingly more accurate in learning List 2 memories compared to simulated control rats [significant main effect of inactivation condition, *F*_(1, 54)_ = 21.18, *p* = 0]. Also, there was a significant main effect of training block [*F*_(2, 54)_ = 61.74*p* = 0], but no interaction between inactivation condition and training block [*F*_(2, 54)_ = 0.09, *p* = 0.92]. In our model, List 1 memories for muscimol rats were initially acquired with activities enabled for all contextual ensembles in DG. As a result, CA3 memory engrams gradually became tuned to DG cells across various contextual ensembles during initial encoding. Although simulated muscimol rats did continue learning for two additional blocks with the top-down contextual bias over DG in place (i.e., with only one appropriate ensemble of DG cells facilitated), we suggest that the distributed projections from across various contextual DG ensembles to CA3 for the List 1 memory engrams were still sufficiently preserved by the time List 2 learning began. During List 2 acquisition in the same context, the activation of the conflicting memory traces in CA3 for simulated muscimol rats was not as strong as that for simulated control rats because the DG support for pattern completion in CA3 was critically shrunk to just one contextual ensemble. In other words, List 2 acquisition underwent relatively weaker interference from previously acquired List 1 memory traces for simulated muscimol rats, explaining the reversal in the encoding dynamics for List 2 between the muscimol and control rats.

## 5. Discussion

The main contribution of this article is its computational and algorithmic explanation for how the hippocampus could employ top-down contextual signals originating in prefrontal cortex (PFC) to reduce interference when encoding and retrieving conflicting associative memories experienced in different contexts. Specifically, we demonstrated that external signals that modulate cell excitability to select a contextually relevant subset of dentate gyrus (DG) neurons play a key role in replicating the rat data on various negative as well as positive effects of inactivating PFC on associative memory formation and recall (Navawongse and Eichenbaum, 2013; Peters et al., 2013). Pre-training the model to learn conflicting memory associations in contexts other than those used in the experiments is the other key element we found necessary to account for the multifarious aspects of the behavioral data. This pre-training qualitatively simulates prior experiences of the adult lab rats, which can conflict with target memories when the contextual bias is suppressed as a result of PFC inactivation. In this regard, even though Experiment 1 of Peters et al. (2013) did not directly manipulate context (see Figures 5, 7), we show that inactivating PFC can lead to additional corollary activation of interfering memory traces from multiple other, previously experienced contexts. Along these lines, Experiment 2 of Peters et al. (2013) and our model simulations (see Figure 8) show that under PFC inactivation, the advantage of a new context in reducing interference while learning a conflicting set of memory associations is lost.

As mentioned in the Introduction section, we define “context” as the functional background with respect to which salient objects in the foreground (comprising an episode) are experienced. Context is not simply a collection of spatial locations that encompass an environment; it is a more holistic construct based on rapidly fusing several relevant spatial and non-spatial cues. Its chief function is to aid in disambiguating the perception of stimuli, recall of memories, and choice of actions (Doboli et al., 2000; Lee and Lee, 2013). Several studies have implicated PFC in rapid extraction of contextual information, which is then leveraged for top-down contextual facilitation of memory-related functions ranging from recognition to recall (Bar, 2004). Moreover, different sub-regions of PFC have been shown to be involved in different aspects of cue-based episodic memory retrieval (Dobbins et al., 2002). This emerging understanding is in concert with the notion that PFC is the source of the external contextual bias to the hippocampus.

Alternative theories posit that contextual information arrives at the hippocampus through the entorhinal cortex (EC) as an explicit input that is associated with other cortical signals coding for salient objects to form an episodic memory (O'Reilly and Rudy, 2001; O'Reilly, 2004). These Complementary Learning Systems models (O'Reilly et al., 2014) perform pattern completion for the AB-AC associative learning task (Barnes and Underwood, 1959) using EC as the gateway for cortical inputs representing list elements and context, and result in the encoding of context and list items as a joint representation within DG. Although the CLS models could, in theory, simulate context-dependent memory retrieval by providing context as one of the recall cues, they would not exhibit contextual clustering of cells within the DG. Very similar list items in different contexts would result in significantly overlapping DG ensembles across contexts, which would be inconsistent with well-known data. In particular, the degree of overlap among sets of DG cells that get activated for various memories within two different contexts is about just 1% (Thompson and Best, 1989; Ramirez et al., 2013). Moreover, the selection of a new ensemble of DG cells can also be triggered by a change in the behavioral task within the same environment (Chawla et al., 2005; Leutgeb et al., 2007; Satvat et al., 2011; Schmidt et al., 2012; Deng et al., 2013). Recent experimental work has examined the role of a local inhibitory circuit in DG between granule cells and hilar perforant path (HIPP) cells that modulates the strength of contextual memories (Raza et al., 2017). In particular, the study found that the mechanism behind the formation of contextual granule cell ensembles in DG involves HIPP cells. To our knowledge, there has been only one computational model (Doboli et al., 2000) that simulates the recruitment and sustenance of contextual ensembles (“latent attractors”) of DG cells, while responding to time-varying inputs from EC. Indeed, as EC inputs change, different cells within the winner DG ensemble become active. In this model, contextual hilar cells within DG are pre-configured to receive excitatory projections from the corresponding granule cells, while they inhibit granule cells configured for the other contexts, and vice versa. Recruitment of an arbitrary ensemble can be achieved by an excitatory perturbation of the appropriate set of DG cells, pointing to control by an external source. This model, however, does not exploit the contextual coding in DG to understand interference-free pattern completion and memory retrieval in an adaptive hippocampus.

It is interesting to note that, in addition to its large size and highly sparse activity compared to other regions of the hippocampus, DG is one of the few brain areas where adult neurogenesis has been reported in mammalian brains including humans (Kuhn et al., 1996). Moreover, the rate at which new DG granule cells are born in adult mice has been shown to covary with the variety and richness of their experiences (Kempermann et al., 1997, 1998). We speculate that the self-regulated birth of new DG cells continuing through adulthood is what enables animals and humans alike to acquire memories in ever-new contexts through their lifespans without catastrophic forgetting of older contextual memories. The effect of neurogenesis in the hippocampus has been examined in computational models that simulate this experience-dependent recruitment of new granule cells and their extended process of maturation resulting in changes in their excitability (Aimone et al., 2009). This could facilitate the use of temporal information as another aspect of the contextual modulation in the encoding process (Rangel et al., 2014). Despite the high excitability of immature DG granule cells, they do not respond broadly to afferent activity from EC due to their much sparser connectivity than mature cells, supporting their role in providing orthogonal representations for encoding new memories that are similar to those previously experienced (Dieni et al., 2016). Another modeling study has investigated the advantageous role of a heterogeneous population of DG granule cells for encoding an increasingly large number of new inputs (Severa et al., 2017). In addition, the reduction of neurogenesis in DG has been experimentally observed when using focal cranial irradiation to eliminate the advantage of a new context for the acquisition of conflicting odor discrimination memories (Luu et al., 2012). While DG has been previously shown to be critical for both the encoding and recall of contextual memories of emotional experiences in rats (Hernández-Rabaza et al., 2008; Bernier et al., 2017), a recent optogenetic study has demonstrated that successful contextual fear conditioning relies on adult-born DG granule cells arising from neurogenesis (Huckleberry et al., 2018).

Depue (2012) reviewed prior literature to develop a conceptual model of prefrontal-hippocampal interactions in which PFC plays the key role of suppressing task-irrelevant memories. Three specific hypotheses for PFC-mediated inhibitory control of cue-triggered memory retrieval were considered (see their Figure 1); namely, (1) “direct inhibition” of specific memory engrams in the hippocampus (Anderson et al., 2004), (2) “reactivation inhibition” of the memory retrieval output of the hippocampus to its neocortical targets including EC (Depue et al., 2007), and (3) “competitive attentional inhibition” by shifting attention to distractor memory engrams in the hippocampus (Wimber et al., 2008). Depue (2012) concluded that prior behavioral and imaging studies related to white bear suppression, directed forgetting, think/no-think tasks provide support only for the direct inhibition and reactivation inhibition hypotheses. While Depue's model relates more to proactive memory suppression than to context-dependent memory retrieval, our hypothesis of PFC inhibitory control to suppress inappropriate contextual ensembles of DG cells is closer to the direct inhibition hypothesis. Large-scale optical imaging of single cells in each hippocampal subfield and the superficial and deep layers of both LEC and MEC will be required to clarify the precise modus operandi of PFC inhibition on memory circuits.

### 5.1. Possible indirect route for pfc bias of hippocampus

No direct mechanism for PFC control of inhibitory bias on DG cells has been identified, but there are some possibilities for an indirect connection. PFC can disynaptically modulate hippocampal cell excitability via hypothalamus and thalamus (Wyss et al., 1979). Engin et al. (2015) showed that tonic inhibition of DG granule cells by GABAergic interneurons, which regulates their excitability, is indeed critical for minimizing interference in the encoding of similar or overlapping memories experienced at different times. Such inhibitory control signals can originate from PFC and arrive at DG by means of hypothalamic supramammillary nucleus (SuMN) (Vertes and McKenna, 2000; Nakanishi et al., 2001). SuMN is connected strongly to DG as well as to several structures that project to the hippocampus, and has been suggested to gate the flow of neural signals within the hippocampus (Vertes, 1992) possibly by modulating cell excitability (Mizumori et al., 1989; Jiang and Khanna, 2006). SuMN has also been shown to key for achieving memory specificity (Xu and Südhof, 2013). Fear conditioning in the absence of SuMN signals to DG would lead to encoding of fear memories across various DG contextual ensembles, resulting in over-generalization to other contexts. While the PFC-SuMN-DG circuit seems pertinent as a mechanism for inhibitory bias on DG, it is unclear how the relatively small number of neurons in the SuMN are capable of biasing the huge number of very specific contextual ensembles that may be activated in the DG.

### 5.2. Testable hypotheses

**Figure 10:**
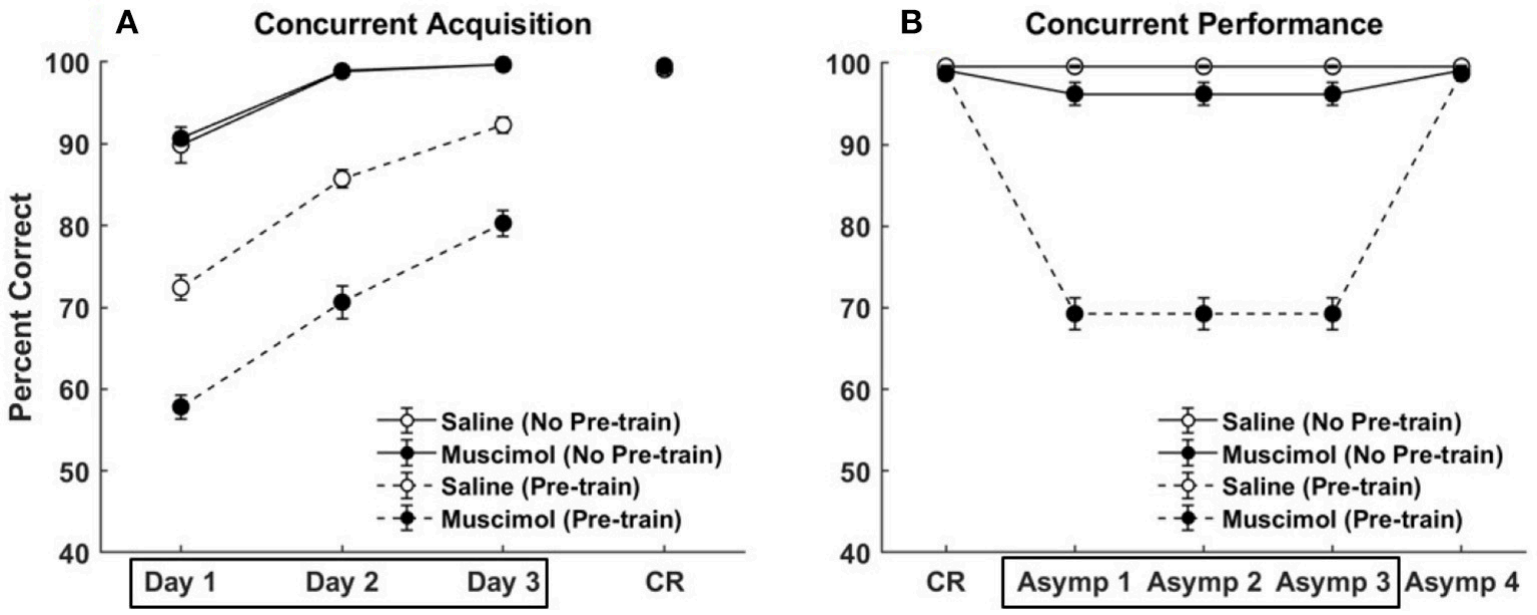
Modeling rat experiment 1A of Peters et al. (2013) without pre-training. Panels **(A,B)** show our model simulation results in the absence of pre-training any conflicting memories, on the effects of PFC inactivation during concurrent acquisition **(A)** and concurrent performance **(B)** of multiple odor discrimination memories. As in Figure 5, the muscimol condition corresponds to PFC inactivation during the first three training blocks (depicted by the box in **A**), and the saline condition is the control. CR on the x-axis in both panels refers to the training block in which rats reached criterion performance. Control rats with saline injection during acquisition received muscimol infusion during the three test blocks following training to criterion (depicted by the box in **B**), but not the muscimol rats. Our model simulation results with pre-training, which are shown in Figure 5, are repeated here with dashed lines for comparison. The error bars correspond to standard error of mean.

**Figure 11:**
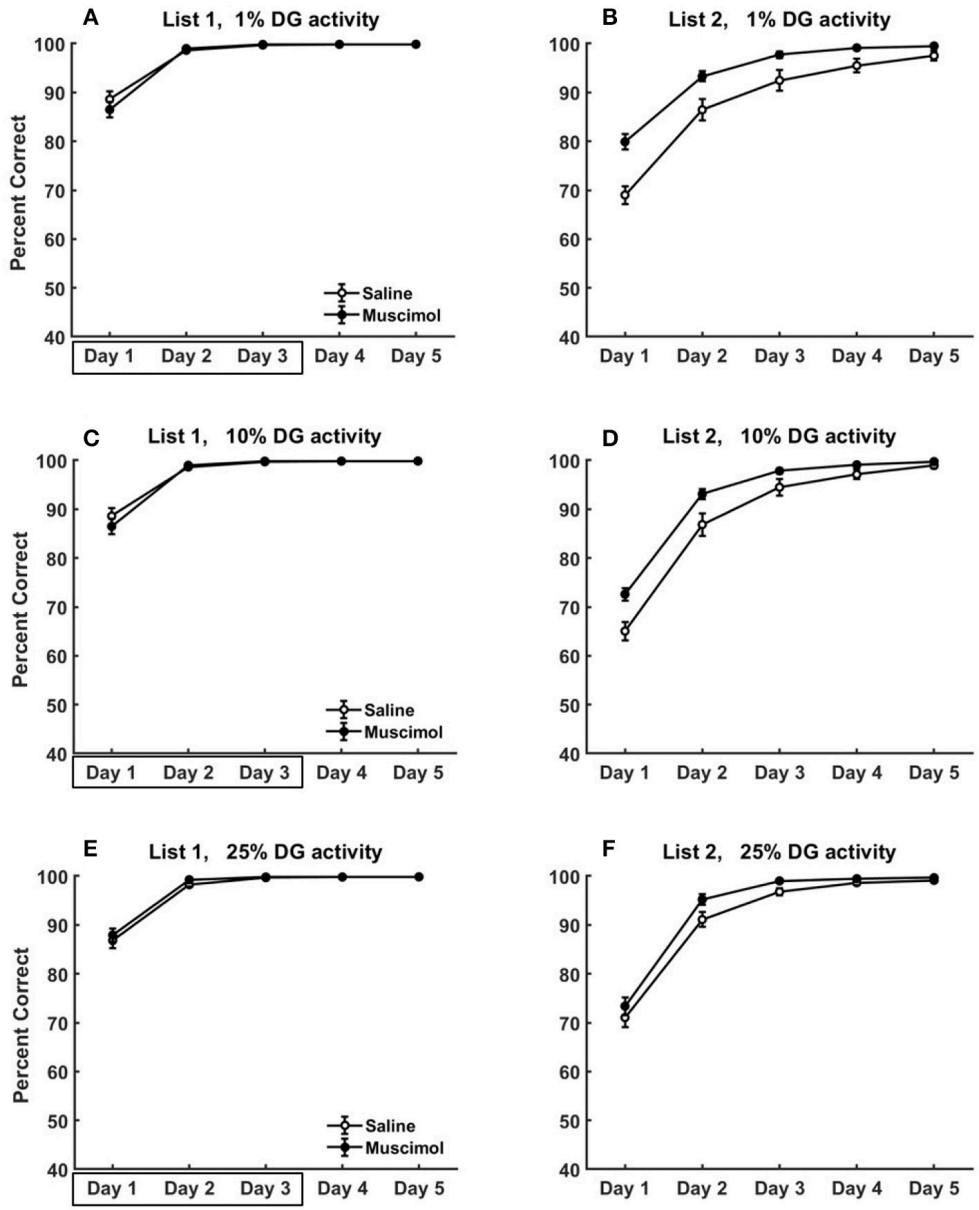
Modeling rat experiment 3 of Peters et al. (2013) by varying the sparseness of activity in DG. The three rows provide our model simulation results in the absence of pre-training any conflicting memories, and at different sparseness levels of activity in DG (top row: *k* = 1%, middle: *k* = 10%, bottom: *k* = 25%), on the acquisition of List 2 memories **(B,D,F)** following the encoding of conflicting List 1 memories **(A,C,E)** in the same context with and without PFC inactivation. Note that as *k* is increased (see Equation 5) for DG, the activities in DG become more dense (i.e., less sparse). As in Figure 9, the muscimol condition corresponds to PFC inactivation during only the first three blocks of List 1 acquisition (depicted by the box in **A,C,E**), and the saline condition is the control. The error bars correspond to standard error of mean.

Future experiments are in order to clarify the precise influences of contextual signals on hippocampal processing in general and DG in particular. In this regard, our mechanistic explanation makes several clear predictions. First, in order to match the data from Experiments 1A, 1B, and 2 of Peters et al. (2013), it was crucial that we pre-train the hippocampus with interfering memories in contexts different than those used to train the target memories in the experiments. This would suggest that PFC inactivation should not affect hippocampal function as assessed in these experiments for animals raised in a single environment, compared to animals with a large number of experiences across multiple environments. In other words, inexperienced animals do not have a storehouse of contradictory associations across different contexts that are unleashed when PFC is inactivated. On the other hand, we predict that the facilitation of List 2 acquisition due to prior PFC inactivation during learning of conflicting List 1 memories, seen in Experiment 3 of Peters et al. (2013), would still hold for these inexperienced rats. This is because the interference from List 1 on the acquisition of List 2 memories would be instantiated within the time course of the experiment. Figures 10, 11 (top row) show quantitative predictions of our model in the absence of pre-training for Experiments 1A and 3 of Peters et al. (2013). As can be seen in Figure 10 with respect to Experiment 1A of Peters et al. (2013), the difference in memory behavior between the saline and muscimol conditions is eliminated during concurrent acquisition [no effect of inactivation condition, *F*_(1, 54)_ = 0.15, *p* = 0.70] and greatly reduced during concurrent performance (from 30.45% drop in Figure 5B to 3.36% drop in Figure 10B). In particular, there was no effect of inactivation condition during concurrent acquisition [*F*_(1, 54)_ = 0.15, *p* = 0.70]. However, as can be seen in Figure 11 (top row) with respect to Experiment 3 of Peters et al. (2013), while the difference in memory behavior between the saline and muscimol conditions is eliminated during List 1 acquisition [no effect of inactivation condition, *F*_(1, 54)_ = 2.52, *p* = 0.12], the facilitation in List 2 acquisition for muscimol rats remains [significant main effect of inactivation condition, *F*_(1, 54)_ = 28.39, *p* = 0]. Furthermore, it seems that the lack of previous conflicting experiences also renders the task of acquiring a list of 8 discrimination memories (namely, List 1) from scratch much easier due to the absence of any implicit interference within the hippocampal network (especially CA3).

We also explored the effects of varying the sparseness of activity in DG on the acquisition dynamics of conflicting List 1 and List 2 memories for Experiment 3 of Peters et al. (2013). These simulations were done in the absence of pre-training any conflicting memories to better isolate the role of sparse activities in DG for this data (see Figure 11). As DG activities become more dense, the pattern separability within DG for similar memories would be expected to be weakened leading to slower memory formation. However, we see no apparent differences in List 1 acquisition across the three sparseness levels (compare List 1 results from the 3 rows in Figure 11). While the memory capacity of our hippocampal network is reduced with more dense DG activities, it is likely much higher than the 8 discrimination memories that are being acquired in the case of no pre-training. Interestingly, the facilitation in List 2 acquisition for muscimol rats seems to exhibit a gradual decrease as sparseness levels in DG are reduced [*k* = 10%: significant main effect of inactivation condition, *F*_(1, 54)_ = 17.89, *p* = 0.0001; *k* = 25%: significant main effect of inactivation condition, *F*_(1, 54)_ = 6.11, *p* = 0.0166]. For the muscimol condition, as the activities in DG become more dense across all contextual ensembles (with increasing *k*), the proportional drop in the absolute number of active DG cells from List 1 acquisition to List 2 acquisition would likely not preclude the pattern completion of List 1 memory traces in the recurrent CA3 network (and thereby in CA1 and ECout) to interfere with the formation of List 2 memories.

Second, the number of active DG cells should increase when PFC is inactivated, because numerous contextual ensembles are enabled due to the lack of top-down inhibition, or cell excitability modulation, from PFC. This can be verified using existing optical sensing technologies to measure c-Fos (or some other immediate-early gene) expression per unit area in DG (Liu et al., 2012; Ramirez et al., 2013). Third, if the mossy fibers from DG to CA3 are lesioned, then all the contextual effects simulated here from the Navawongse and Eichenbaum (2013) and Peters et al. (2013) studies should be eliminated or greatly reduced. In particular, these animals will exhibit significant impairments in behavioral tasks that require distinguishing similar or overlapping memories acquired in different contexts.

## 6. Conclusion

The new understanding of how PFC exerts top-down control of hippocampal memory circuits can be leveraged to causally enhance memory performance. Prior rat data suggests increased top-down attention to the context in which events occur improves the long-term stability of memories (Kentros et al., 2004; Muzzio et al., 2009a,b). Moreover, a number of human studies demonstrating declarative memory enhancement by transcranial current stimulation (tCS) during encoding have targeted the PFC (Javadi and Walsh, 2012; Javadi and Cheng, 2013). Interestingly, transfer learning, where a schema learned in one context is applied to a different context, has been shown to be inversely related to functional connectivity between PFC and hippocampus (van Kesteren et al., 2012; Gerraty et al., 2014). When contextual biasing signals from PFC on DG are weak, memories learned in one context generalize to other contexts. Stronger contextual signals from PFC lead to greater memory specificity during encoding, resulting in more stable memories. In summary, our memory model provides a computational account for the top-down contextual modulation of encoding and recall processes in the hippocampus by postulating contextual recruitment of independent pools of DG cells that critically depends on normal PFC operation.

## Author contributions

PP conceived the study. MH and PP performed the simulation experiments. MH, PP, and RB analyzed the data. PP and MH wrote the paper.

### Conflict of interest statement

The authors were employed by HRL Laboratories, LLC, and have a pending patent application on a contextually biased memory system. The authors declare that the research was conducted in the absence of any commercial or financial relationships that could be construed as a potential conflict of interest.
